# Ovarian hormone loss modifies the nucleic acid cargo of circulating extracellular vesicles and skeletal muscle metabolism after acute exercise in rats

**DOI:** 10.1113/EP094085

**Published:** 2026-08-10

**Authors:** Veera Puumalainen, Anni Maja, Tia‐Marje Korhonen, Tuuli A. Nissinen, Eero Hulkko, Janne A. Ihalainen, Maarit Lehti, Sira Karvinen

**Affiliations:** ^1^ Faculty of Sport and Health Sciences University of Jyväskylä Jyväskylä Finland; ^2^ Department of Biological and Environmental Science University of Jyväskylä Jyväskylä Finland; ^3^ Nanoscience Center Department of Biological and Environmental Science University of Jyväskylä Jyväskylä Finland; ^4^ Nanoscience Center Department of Chemistry University of Jyväskylä Jyväskylä Finland; ^5^ Gerontology Research Center University of Jyväskylä Jyväskylä Finland; ^6^ Department of Molecular and Integrative Biosciences Faculty of Biological and Environmental Sciences University of Helsinki Helsinki Finland

**Keywords:** extracellular vesicles, high‐density lipoprotein, menopause, oestrogen deficiency, ovariectomy, Raman spectroscopy, small non‐coding RNA

## Abstract

Loss of ovarian hormones (i.e., menopause) leads to negative effects on metabolic health. While exercise offers significant benefits, it seems to be insufficient to completely reverse these changes. The mechanisms by which exercise conveys the effects throughout the body are still poorly understood. Extracellular vesicles (EVs) are released into circulation during exercise. EVs carry small non‐coding RNAs (sRNAs), such as microRNAs (miRs), which are proposed as mediators of the effects of exercise. We have previously shown that the miR response to acute exercise of EVs and high‐density lipoprotein (HDL) is diminished in postmenopausal women with low oestrogen levels, which we were able to replicate here also in a rat model. In this study, we examined the effect of loss of ovarian hormones and acute exercise in the sRNA cargo of EV and HDL particles in female rats. We show for the first time that loss of ovarian hormones affects specifically the nucleic acid cargo of circulating EVs. We further show that the oestrogen responsive miRs regulate anaerobic glycolytic pathway. Finally, we demonstrate that the loss of ovarian hormones leads to higher anaerobic energy production during an acute bout of exercise, implicating an inferior ability to sustain aerobic energy production during exercise.

## Abstract (highlights)

- **What is the central question of this study?**
  How does the loss of ovarian hormones alter the sRNA cargo of circulating extracellular vesicles (EVs) and high‐density lipoprotein particles in response to acute exercise, and do these molecular changes help explain impaired metabolic regulation and a shift toward anaerobic energy production?
- **What is the main finding and its importance?**
  Ovarian hormone loss specifically alters the nucleic acid cargo of circulating EVs and exercise‐responsive microRNAs linked to glycolysis. This is associated with greater reliance on anaerobic energy production during exercise, suggesting a mechanism by which menopause may alter metabolic flexibility in response to exercise.

## 1 INTRODUCTION

Loss of ovarian hormones (i.e., menopause) is associated with several negative health effects, such as increased adipose tissue mass and higher risk for metabolic diseases including type 2 diabetes (Clegg et al. 2017; Juppi et al. 2022). While exercise appears to ameliorate these changes, it cannot completely reverse them (Hyvärinen, et al. 2022; Karvinen et al. 2019). We have previously shown that menopause diminishes the acute exercise response of circulating extracellular vesicles (EVs) in postmenopausal women (Karvinen et al. 2023). Yet it is not known how this change in systemic signalling may affect the response to exercise at tissue level.

Withdrawal of ovarian hormones by ovariectomy (OVX) in rodents is widely used to mimic the effects of menopause in the female body. The deficiency of oestrogens, predominantly oestradiol (E2), is the main hormonal contributor to the menopause‐related physiological changes (reviewed in Camon et al. 2024). Loss of ovarian hormones in women and female rats is associated with increased body weight and adipose tissue mass, as well as a decline in metabolic health (Asarian & Geary, 2002; Juppi et al. 2022; Lee et al. 2024). Furthermore, similarly to menopause, the loss of ovarian hormones has been associated with decreased physical activity levels in rodents (Chen et al. 2014; Mäkinen et al. 2023; Pettee Gabriel et al. 2015). We have previously shown that loss of ovarian hormones decreases also the maximal running capacity in female rats (Lee, et al. 2024). Accordingly, utilizing an OVX model allows for the investigation of the interplay of ovarian hormones and exercise at the tissue level.

While the primary operators of exercise are the skeletal muscles and the cardiorespiratory system, its effects are systemic, influencing the entire body. In recent years, it has been demonstrated that exercise induces release of EVs (Frühbeis et al. 2015; Vechetti, et al. 2021; Whitham, et al. 2018) and high‐density lipoprotein (HDL) particles (Karvinen et al. 2023; Palazón‐Bru, et al. 2021) into the circulation. EVs and HDL act as information carriers in systemic signalling, carrying for example lipids, proteins and small non‐coding RNAs (sRNAs), such as microRNAs (miRs) (Théry et al. 2002; Vickers & Remaley, 2014). MiRs, including those carried by EVs, have been implicated in regulation of skeletal muscle pathways, coordinating, for example, growth, development and metabolism (Liu & Dong, 2025). For example, EV‐carried miRs have been shown to regulate expression of genes related to differentiation in mouse skeletal muscle myoblasts (Forterre et al. 2013). However, the role of EVs and HDL in conveying the positive effects of exercise is still poorly understood.

EVs are membrane‐bound particles which are released by all types of cells. EVs have been shown to facilitate cell‐to‐cell communication (Kalra et al. 2016; Mathivanan et al. 2010) and to act as inter‐organ communication mediators, also in response to exercise (Vechetti et al. 2021; Whitham & Febbraio, 2016). Like EVs, also HDL transports sRNAs to recipient cells (Karvinen et al. 2023; Vickers et al. 2011). The capability of HDL to transport sRNAs can possibly explain, at least partially, the mechanism by which HDL is able to act as a modulator of recipient cell functions. The sRNA profile of EV cargo differs from that of HDL (Karvinen et al. 2023), suggesting that these communication mediators have complementary but independent mechanisms for sRNA transport. Understanding systemic signalling allows us to uncover the molecular mechanisms underlying the effects of exercise on health.

sRNAs are not translated into proteins yet regulate numerous central cellular processes. sRNAs have been shown to respond to exercise (Chen et al. 2012; Iguchi et al. 2010; Nielsen et al. 2010; Quinn & Chang, 2016). Of sRNAs, the functions of miRs have been studied most extensively (Gomes et al. 2018). Circulating miRs are shown to deliver signals from donor cells to recipient cells both in vitro and in vivo (Iguchi et al. 2010). The main function of miRs is to negatively regulate protein translation by affecting the stability of messenger RNA (Huntzinger & Izaurralde, 2011). miRs are implicated to play a role, for example, in metabolism of lipids and glucose (Poy et al. 2007; Rottiers & Näär, 2012), inflammation (Das & Rao, 2022), and coordinating ageing‐related biological pathways (Inukai & Slack, 2013). Extensive studies have revealed that the circulating miR profile changes in response to exercise (Barber et al. 2019; Li et al. 2020; Nielsen, et al. 2014).

We have previously shown that the acute miR response to exercise in EVs is diminished in post‐menopausal women (Karvinen et al. 2023). Here, we examined this further with female rats, which enables studying the phenomenon on a tissue level. We utilized Raman spectroscopy to study the effect of loss of ovarian hormones, induced by ovariectomy (OVX), on the EV molecular cargo. Furthermore, we investigated the signalling pathways affected by ovarian hormone responsive miRs. Based on the findings from the pathway analysis, we performed supplementary tests to determine how the loss of ovarian hormones influences anaerobic energy production. Since oestrogens regulate mitochondrial function and enhance oxidative phosphorylation (reviewed in Klinge, 2017), we hypothesized that OVX would lead to decreased aerobic energy production, leading to higher dependency on anaerobic energy production.

## 2 METHODS

### 2.1 Ethical approval

The study has received approval from national Project Authorization Board (ELLA, Finland, permit number ESAVI/4209/2021) and was conducted in accordance with the *Principles of Laboratory Animal Care* (NIH publication no. 85‐23, revised in 1985) and the European Commission Directive 2010/63/EU. Additionally, this research complies with the animal experiment policies of the journal.

### 2.2 Animals

**FIGURE 1:**
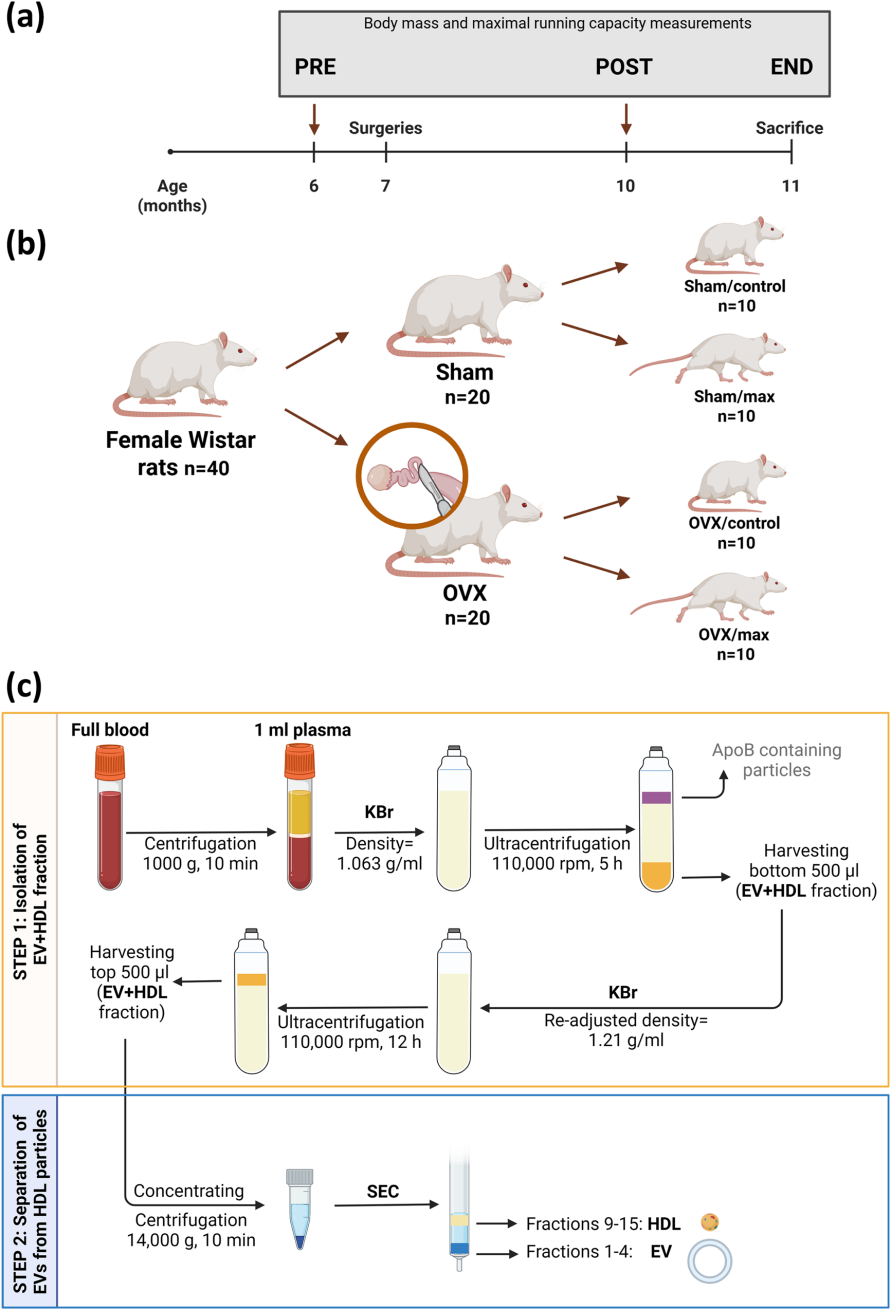
Schematic representation of the study design. (a) Timeline of the study. Rats were matched for body weight and maximal running capacity before dividing them into groups. (b) 40 female Wistar rats were divided to two groups: ovariectomy (OVX), and sham surgery without the removal of ovaries (sham). Groups were further divided so that half of the rats performed a maximal running test to induce an acute exercise stimulus. (c) Flowchart of EV and HDL particle isolation protocol. Step 1: EVs and HDL were isolated based on density; Step 2: EVs were separated from HDL particles based on size. ApoB, apolipoprotein B; EV, extracellular vesicle; HDL, high‐density lipoprotein; KBr, potassium bromide; OVX, ovariectomy; SEC, size‐exclusion chromatography.

The study sample consisted of 40 female Wistar Hans rats, which correspond to a representative subset of study design reported previously by us (Figure 1 a,b; Lee et al. 2024). Rats were purchased from Envigo (Indianapolis, IN, USA, bred and shipped from the Netherlands), and arrived at the animal facilities of University of Jyväskylä at the age of 13–15 weeks. All rats were housed in pairs in an environment‐controlled facility (12/12 h light–dark cycle, 22°C) and received water and oestrogen‐free rodent feed (2019X, Envigo) ad libitum. Half of the rats (*n* = 20) underwent ovariectomy (OVX), resulting in the loss of ovarian hormones, and the other half (*n* = 20) was predisposed to sham surgery without the removal of ovaries. The groups were matched for body mass and maximal running capacity so that these two groups did not differ in these two study variables (Table 1). To ensure that the rats were fully grown adults prior to the surgeries, the surgeries were performed at the age of 7 months (Figure 1a). The OVX and sham groups were further divided into two groups, one performing maximal running test (max) to induce an acute exercise stimulus, and the other serving as a control. Hence, the resulting groups were sham/control, sham/max, OVX/control and OVX/max (*n* = 10 per group, Figure 1b). Rats in the sham group were euthanized at pro‐oestrus (determined by cytology sample) to ensure that they were in the highest possible systemic oestrogen state at the time samples were collected (Lee et al. 2024).

**TABLE 1:** Characteristics of the study groups.

					*P*		
	Sham/control (*n* = 10)	Sham/max (*n* = 10)	OVX/control (*n* = 10)	OVX/max (*n* = 10)	Sham vs. OVX	Sham/control vs. sham/max)	OVX/control vs. OVX/max)
Body mass (g)							
PRE	276.3 (34.0)	280.3 (37.2)	298.4 (42.2)	286.8 (23.8)	0.196	0.805	0.459
POST	312.4 (44.2)	309.1 (41.2)	364.2 (50.3)	359.7 (35.5)	**<** **0.001**	0.865	0.820
END	315.5 (46.6)	315.0 (48.4)	373.0 (59.4)	363.0 (36.7)	**0.001**	0.981	0.657
Total muscle mass^†^ (g)	2.58 (0.29)	2.67 (0.30)	2.75 (0.27)	2.67 (0.23)	0.311	0.473	0.472
Total muscle mass^†^ (% of body mass)	0.82 (0.07)	0.85 (0.06)	0.75 (0.08)	0.74 (0.03)	**<** **0.001**	0.303	0.656
Retroperitoneal fat mass (g)	5.56 (2.57)	5.51 (2.94)	9.82 (4.33)	10.71 (2.46)	**<** **0.001**	0.971	0.580
Uterine mass (g)	0.69 (0.17)	0.71 (0.10)	0.13 (0.02)	0.15 (0.08)	**<** **0.001** ^a^	0.729	0.529^a^
Liver mass (g)	9.15 (0.97)	9.16 (1.30)	8.70 (1.83)	7.96 (0.70)	**0.047**	0.982	0.243
Maximal running capacity (m)							
PRE	395.3 (116.8)	378.6 (178.6)	363.1 (159.7)	373.3 (153.0)	0.694	0.529^a^	0.886
POST	410.8 (114.7)	395.2 (164.5)	327.7 (176.4)	301.0 (87.9)	**0.009** ^a^	0.631^a^	0.853^a^
END		275.3 (86.5)		238.8 (77.7)	0.334		

^a^Mann–Whitney *U*‐test, bold signifies *P *< 0.05. ^†^Calculated sum of average muscle mass (left and right) hindlimb muscles (including gastrocnemius, extensor digitorum longus, tibialis anterior, and soleus) was used to represent the total muscle mass.

### 2.3 Maximal running capacity test

Rats were tested for their maximal running capacity similarly as described earlier (Lee et al. 2024). Briefly, a speed‐ramped treadmill running test (15° slope, initial velocity of 10 m/min, increased 1 m/min every 2 min) was performed PRE and POST the intervention, and for max groups immediately before euthanasia (Figure 1a). First, rats were habituated to running on the treadmill with three different sessions lasting for 10 min with a low velocity (< 10 m/min). Maximal running test was repeated three times with at least 1 day of recovery in between. The best result of the three trials [maximal running distance (m)] was considered the maximal running capacity, except for the test performed immediately before euthanasia for the max groups, where the test was performed only once.

### 2.4 Tissue and blood sampling

At the end of the study, rats were fasted for 2 h and euthanized with carbon dioxide followed by heart puncture. Rats, that performed maximal running test were euthanized within 5 min after the test. Tissue samples (hindlimb muscles, liver and adipose tissue) were collected, weighed and snap frozen in liquid nitrogen and stored at −80°C until further analyses. Of the hindlimb muscles, gastrocnemius, extensor digitorum longus, tibialis anterior and soleus were used to calculate average muscle mass to compare different study groups (Table 1), whereas only gastrocnemius was used for miR target protein analyses by western blot. Liver was collected for miR target protein analyses. Adipose tissue was used to compare the fat mass between study groups (Table 1). Of the adipose tissue deposits, only retroperitoneal adipose tissue was collected and weighed because of its more distinct location and uniform composition compared to visceral (i.e., omental) and/or ovarian adipose tissue deposits. Plasma (EDTA) was separated from the whole blood by 15 min incubation at RT, followed by centrifugation (1000 *g*, 10 min at RT). Plasma was stored as 200 µL aliquots at −80°C.

### 2.5 Isolation of EVs and HDL

The isolation of EVs and HDL was carried out similarly as described before (Karvinen et al. 2023). The workflow of isolation and separation of HDL particles and EVs from plasma is depicted in Figure 1c.

EV and HDL‐containing fractions were isolated by sequential ultracentrifugation, in which the density was adjusted by potassium bromide (KBr) to separate lipoproteins by density difference (Havel et al. 1955). Plasma samples (1000 µL) were thawed, and their volume was adjusted to 2 mL with sterile filtered (0.2 µm) phosphate‐buffered saline (PBS) and density to 1.063 g/ml and ultracentrifuged at 657,000 *g* at + 4°C, (Beckman Coulter, 110 TLA rotor, Brea, CA, USA) for 5 h in 3.2 mL ultracentrifuge tubes (Beckman Coulter, cat. 362333). The bottom 500 µL was collected, volume adjusted to 2 mL and density readjusted to 1.21 g/ml and ultracentrifuged (657,000 *g*, + 4°C) for 12 h. The EV and HDL‐containing top 500 µL fraction was collected and stored at −80°C. Ultracentrifugation results in fraction containing both EVs and HDL, and therefore size‐exclusion chromatography (SEC) was performed to separate EVs from HDL. Prior to SEC, the ultracentrifuged HDL and EV fraction was concentrated by centrifugation (14,000 *g*, +4°C, Heraeus Fresco 17 centrifuge, Osterode am Harz, Germany) with Amicon® Ultra centrifugal filters (100 kDa, cat. UFC5100, Merck, Carrigtwohill, Co. Cork, Ireland) according to the manufacturer's instructions. Concentrates were stored at −80°C until SEC.

EVs and HDL were separated by SEC (IZON qEV1, 35 nm, IC1‐35, Lyon, France). Concentrate containing HDL and EVs was thawed on ice. The SEC column was operated as instructed by the manufacturer at room temperature and in the upright position. To reach recommended sample volume of 1 mL, sterile, filtered (0.2 µm) PBS (pH 7.4) was added to the concentrated sample. One millilitre of concentrated sample was loaded onto the column and 4.7 mL void volume was discarded. Then, 16 fractions were collected, 700 µL each. EVs were collected into fractions 1–4 according to the manufacturer's instructions. From SEC fractions, EVs and HDL were concentrated the same way as prior to SEC.

Throughout the protocol, the EV and HDL‐containing samples were kept in low‐binding tubes (low protein binding microcentrifuge tubes, Thermo Scientific, Rockford, IL, USA, cat no. 90410) when applicable to reduce the loss of particles by adhesion to the microcentrifuge tubes.

### 2.6 Validation of EV and HDL isolation

**FIGURE A1:**
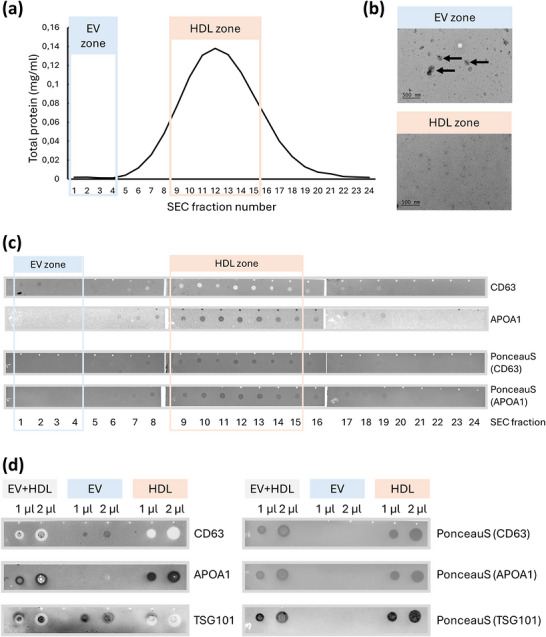
EV and HDL particles enrich in separate fractions. (a) Total protein (BCA) analysis of SEC fractions 1–24 from a representative EV+HDL fraction from plasma. (b) Representative EM images of EV and HDL fractions isolated from plasma. (c) Dot blot verification of enrichment of EVs into SEC fractions 1–4 with CD63 antibody and HDL particles into SEC fractions 9–15 with APOA1 antibody with corresponding total protein blots stained with PonceauS from a representative EV and HDL fractions isolated from plasma. (d) Fractions EV + HDL, EV and HDL separated via SEC and visualized with 1 and 2 µL sample with CD63, APOA1 and TSG101 antibodies (left panel) and corresponding total protein blots stained with Ponceau S (right panel) from representative EV and HDL fractions isolated from plasma. EV, extracellular vesicle; HDL, high‐density lipoprotein; SEC, size‐exclusion chromatography.

The EV zone was located in fractions 1–4 and the HDL zone was located between SEC fractions 9 and 15, which was confirmed by bicinchoninic acid assay (BCA) (Thermo Fisher Scientific, Waltham, MA, USA) with automated instrument (KoneLab, Thermo Scientific, Vantaa, Finland), dot blot and electron microscopy (EM) (Appendix Figure A1).

The amount of protein in the EV samples could not be visualized by traditional western blot (data not shown), so a dot blot assay was performed by pipetting 1 µL of sample directly onto nitrocellulose membrane. For total protein visualization, Ponceau S staining was used, whereas for EVs and HDL, antibodies for CD63 (1:500; cat. no. ab59479, Abcam, Cambridge, UK), APOA1 (1:10,000; cat. no. ab52945, Abcam), and TSG101 (undiluted; cat. no. ab125011, Abcam) were used. CD63 is a transmembrane protein enriched in EVs (Escola et al. 1998), TSG101 is a cytosolic protein expressed in EVs, whilst APOA1 is enriched especially in HDL, but is detected also in EVs (Karimi et al. 2018). Total protein and dot blot confirmed that HDL was enriched in fractions 9–15.

Samples containing EVs and HDL separately (the fractions after SEC) were prepared for EM as described previously (Karvinen et al. 2020; Puhka et al. 2017). Briefly, EV and HDL samples were loaded on 200 mesh grids, fixed with 2% paraformaldehyde (PFA) solution, stained with 2% neutral uranyl acetate, and embedded in a uranyl acetate and methyl cellulose mixture (1.8/0.4%). Samples were viewed with transmission EM using a Jeol JEM‐1400 (Jeol Ltd, Tokyo, Japan) operating at 80 kV. Images were taken with a Gatan Orius SC 1000B CCD‐camera (Gatan Inc., Pleasanton, CA, USA).

### 2.7 Raman spectroscopy

The molecular composition of the EV cargo was studied via Raman spectroscopy from pooled, representative samples of the study groups (*n* = 6 per group). For this purpose, EVs were isolated from plasma using SEC similarly as described in Section 2.5 (Isolation of EVs and HDL). EV samples were analysed using Raman spectroscopy (Nicolet DXR Raman Microscope, Thermo Fisher Scientific, Rockford, IL, USA) equipped with a diode‐pumped solid‐state laser operating at 532 nm following a previously described protocol (Gualerzi et al. 2019). Briefly, 5 µL of EV suspension was deposited on a gold slide. All the measurements were performed on the air‐dried drop with ×50 objective, 900 lines/mm diffraction grating, 700 µm spot size, and confocal mode (50 µm pinhole) in the spectral range of 400−3581 cm^−1^. For each group, five measurements were collected and used for the analysis.

The Raman spectra were processed using MATLAB (MathWorks, Natick, MA, USA) by fitting with two linear lines to subtract the background from the signal. Thereafter, the signal was normalized to the highest lipid peak. To calculate the nucleic acid to lipid (NA/L) and protein to lipid (P/L) ratios as described previously (Mihály et al. 2017), we first calculated the average of each molecular class (nucleic acid band, 720–800 cm^−1^; amide I protein band, 1600–1690 cm^−1^; and lipid‐related band, 2750–3040 cm^−1^). Unfortunately, we were unable to assess the molecular cargo of HDL by Raman spectroscopy.

### 2.8 Small RNA extraction and sequencing

Before RNA extraction, the SEC fractions containing EVs and HDL particles were concentrated similarly as described in Section 2.5 (Isolation of EVs and HDL). RNA was extracted using miRNeasy Serum/Plasma Kit (Qiagen, Hilden, Germany, cat. no. 217184) according to the manufacturer's instructions. Thereafter, 10 µL of RNA isolate was shipped to Novogene (Planegg, Germany) for exosomal small RNA library preparation and sequencing.

### 2.9 Raw sRNA data processing and alignment

Clean reads were received from Novogene in FASTA format. Reads shorter than 20 nucleotides were excluded. For the analysis of miRs, the reads were trimmed to 22 bp using a FastX‐Toolkit (Gordon & Hannon, 2010; available online at: http://hannonlab.cshl.edu/fastx_toolkit). The alignment was done using Bowtie (Langmead et al. 2009). No reverse complement option was used. Only one best alignment for a read was used as output, even if there were multiple possible alignments. miRs were aligned to miRBase version 22 (Kozomara et al. 2019), ribosomal RNA (rRNA)‐derived sRNAs (rDRs) were aligned to the full set of rat rRNA (downloaded from RNAcentral in February 2025) and transfer RNA (tRNA)‐derived sRNAs (tDRs) were aligned to the high confidence tRNA gene set from GtRNAdb (Chan & Lowe, 2016). For miR sequences, two mismatches were allowed; for tDR sequences, one mismatch was allowed; and for rDR sequences, no mismatches were allowed.

### 2.10 TIGER pipeline analysis of sRNA

The sRNA species in EV and HDL particles were analysed with the Tools for Integrative Genome analysis of Extracellular sRNAs (TIGER) pipeline (Allen et al. 2018). This analysis categorizes the sRNA species by their origin by utilizing genome and database alignments (Figures 3, 4).

### 2.11 Differential expression analysis on sRNAs

Differential expression (DE) analyses were done as described earlier (Karvinen et al. 2023) using DESeq2 R‐package in R‐program (v4.3.1) for EV and HDL samples (*n *= 10 per group each). Samples with fewer than 10 sRNA (miR, rDR or tDR) counts in at least seven samples in a subgroup were excluded from the analysis.

### 2.12 miR target analysis and signalling pathway heatmap

To elucidate the possible functional roles of EV‐ and HDL‐carried miRs that were differentially expressed in response to exercise, the possible interactions of miRs and mRNAs were examined by miRPath v3.0 (Vlachos et al. 2015), using Kyoto Encyclopedia of Genes and Genomes (KEGG) pathway analysis with pathways union option. In miRPath analysis, microT threshold was set to 0.08, and a *P*‐value of 0.06 was used to avoid excluding several essential exercise‐related miR signalling pathways. Central pathways to energy production were analysed further, and target proteins of differentially expressed miRs were examined from these pathways.

### 2.13 Western blot analysis of the miR target proteins

Western blot analysis was performed to measure the target protein levels of the differentially expressed miRs detected from the EV and HDL fractions. The analysed proteins were mammalian target of rapamycin (mTOR), insulin receptor substrate 2 (IRS2) and pyruvate kinase L/R (PKLR), which were chosen based on the miR target analysis via miRPath v3.0 (Section 2.12 miR target analysis and signalling pathway heatmap). It has been noted that EVs released after exercise tend to localize to liver tissue (Whitham et al. 2018), and therefore the expression of target proteins in liver was investigated. Given its role in exercise, it was reasoned to analyse also muscle protein levels. In addition, both tissues are crucial for energy metabolism (Whitham et al. 2018).

First, the liver and gastrocnemius muscle samples (30 mg) were homogenized in buffer solution [20 mM HEPES (pH 7.4), 1 mM EDTA, 5 mM EGTA, 10 mM MgCl_2_, 100 mM β‐glycerophosphate, 1 mM Na_3_VO_4_, 2 mM dithiothreitol, 1% NP‐40, 0.2% sodium deoxycholate (C_24_H_39_O_4_Na), and 3% protease and phosphatase inhibitor cocktail (Thermo Scientific, cat. no. 78443)] in TissueLyser II (Qiagen) for 2 × 2 min, 30 Hz. Next, the homogenates were kept on a sample rocker for 30 min at +4°C and centrifuged at 10,000 *g* at +4°C for 10 min. Total protein was determined by bicinchoninic acid (BCA) assay with an automated instrument (KoneLab, Thermo Scientific, Vantaa, Finland) and diluted to 2 µg/µL. Laemmli buffer was added to the samples, and the samples were heated for 10 min to linearize the proteins. Separation was done by SDS‐PAGE for 30–40 min at 270 V on a Criterion electrophoresis cell (Bio‐Rad Laboratories, Hercules, CA, USA) on 4–20% gradient gels (Criterion™ TGX Stain‐Free™ Precast Gel, Bio‐Rad, cat. no. 5678094). Proteins were transferred onto nitrocellulose membrane in Turbo blotter (Trans‐Blot Turbo Blotting System, 170–4155, Bio‐Rad). Thereafter, the membrane was blocked for 2 h at room temperature in a commercially available blocking buffer [Intercept® (PBS) Blocking Buffer, LI‐COR Biosciences (Lincoln, NE, USA), cat no. 927–70001]. Then the membrane was incubated overnight at +4°C in primary antibody solution to measure target proteins: mTOR (2983S, Cell Signaling Technology, Danvers, MA, USA, RRID:AB_2105622), IRS2 (ab134101, Abcam, RRID: AB_2810948) and PKLR (ab137787, Abcam), all at dilution 1:1000 in 1:1 blocking buffer and Tris‐buffered saline (TBS). After the incubation, membrane was washed in TBS with Tween 20 (TBS‐T) and incubated in suitable secondary antibody diluted in 1:1 blocking buffer and TBS‐T for 2 h at room temperature and washed again in TBS‐T. Fluorescence images were obtained with the ChemiDoc MP™ Imaging System together with Image Lab™ Touch software (version 2.4.0.03, Bio‐Rad Laboratories). Proteins were quantified using Image Lab software (version 6.1.0). The blot lanes were first normalized to blot average, and the individual bands were subsequently normalized to corresponding lane averages. The results are expressed as arbitrary units (AU).

### 2.14 Lactate dehydrogenase enzyme activity measurement

To assess the anaerobic energy production in skeletal muscle, the same gastrocnemius muscle homogenates as utilized for the western blot analysis were subjected to lactate dehydrogenase (LDH) analysis (Thermo Fisher Scientific, cat. no. 981906). The muscle homogenates were diluted 1:20 into milliQ H_2_O and the LDH activity was measured from single samples (*n* = 10/group) via Indiko Plus (Thermo Fisher Scientific, Vantaa, Finland).

### 2.15 Statistics

Statistical analyses were conducted using IBM SPSS Statistics (version 28.0.1.1; IBM Corp., Armonk, NY, USA). The normality was assessed by the Shapiro–Wilk test followed by Levene's test. When data were normally distributed, group comparisons were made with Student's *t*‐test, and when the data did not meet the criterion for normal distribution, the Mann–Whitney *U*‐test was used. Two‐way ANOVA was used for assessing the main effects of OVX, acute exercise and their interaction. A *P*‐value < 0.05 was regarded as statistically significant. Extreme outliers (>3 × interquartile range) were excluded from the analysis.

## 3 RESULTS

### 3.1 The loss of ovarian hormones increased body fat mass and reduced maximal running capacity

As OVX surgery is known to increase body mass and decrease physical activity level, the rats were matched for their body mass and maximal running capacity before dividing them into sham and OVX groups (PRE, *P* ≥ 0.196, Table 1, Figure 1). Furthermore, the sham and OVX groups were divided into control and max groups, matched for body mass and maximal running capacity (PRE, *P* ≥ 0.459, Table 1, Figure 1).

Within‐group (OVX or sham) comparisons confirmed that the subgroups (max vs. control) did not differ in the studied variables (*P* ≥ 0.243, Table 1). Post‐intervention sham versus OVX comparisons revealed significant differences in several variables (Table 1). OVX rats exhibited higher body mass and retroperitoneal fat mass, as well as lower relative muscle mass than sham (*P *< 0.001), while absolute muscle mass did not differ between the groups (*P* = 0.311, Table 1). Uterine mass was significantly reduced in OVX, confirming successful ovariectomy surgeries (*P *< 0.001, Table 1).

### 3.2 The loss of ovarian hormones changed the nucleic acid‐to‐lipid ratio in EVs in response to acute exercise

**FIGURE 2:**
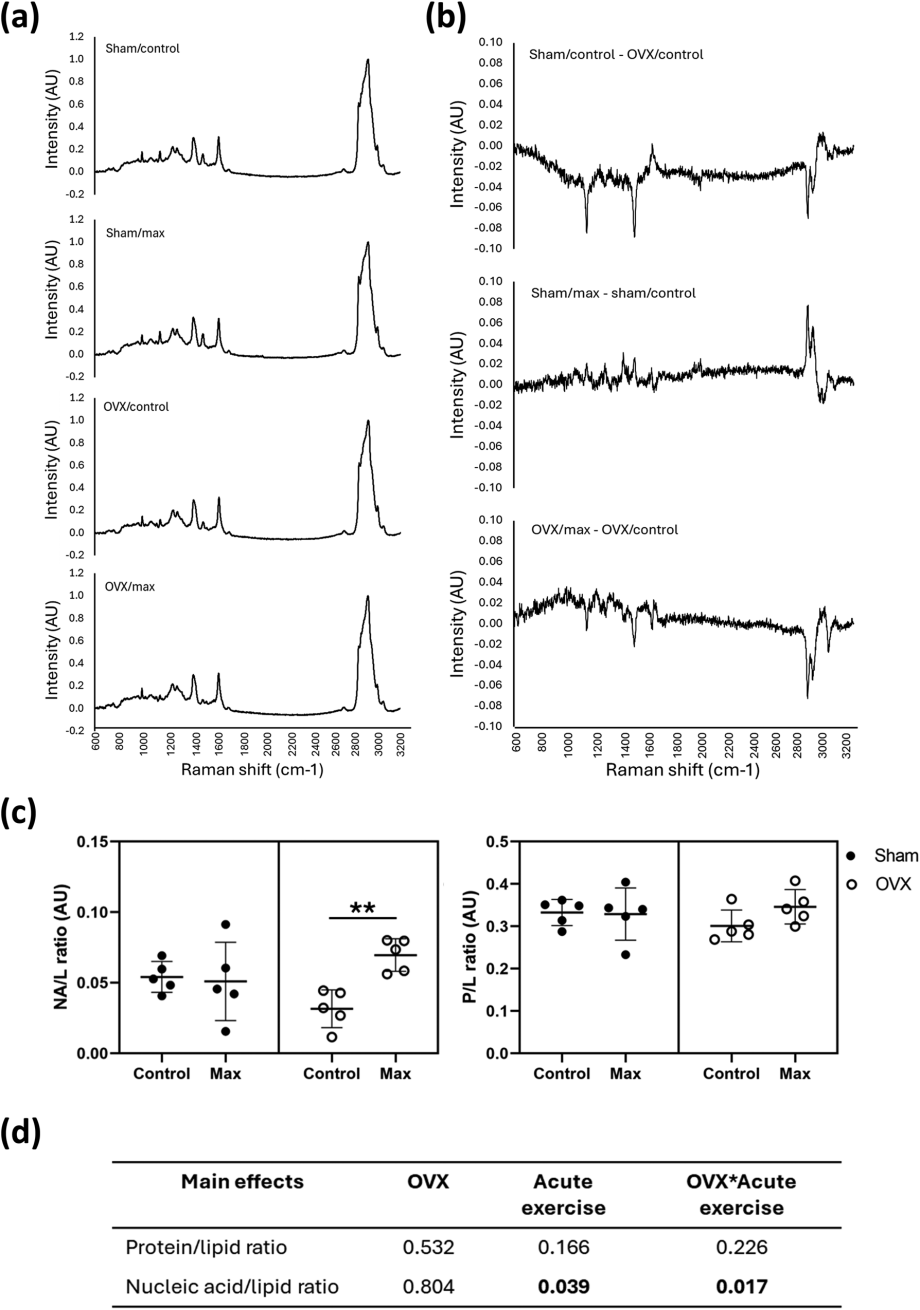
Loss of ovarian hormones (induced by OVX) changes specifically the nucleic acid cargo of EVs in response to exercise. (a) Full Raman spectra exhibit consistent peak patterns across the groups. (b) Subtraction spectra display differences between groups. (c) OVX and acute exercise affect significantly the nucleic acid‐to‐lipid (NA/L) ratio, whereas protein to lipid (P/L) ratio did not display changes. (d) Main effects of OVX, acute exercise and the interaction of OVX and acute exercise on P/L and NA/L ratio (*P*‐values). Acute exercise and the interaction of OVX and acute exercise (OVX × Acute exercise) affect significantly the NA/L ratio, whereas no effects were seen on the P/L ratio. L, lipid; NA, nucleic acid; OVX, ovariectomy; P, protein.

The Raman spectra exhibited consistent peak patterns across all groups, suggesting uniform quality among the samples (Figure 2a). However, despite the Raman spectra similarity between groups, the spectral subtraction graphs showed differences between the groups (Figure 2b). We observed a significant effect of acute exercise and an interaction of OVX and acute exercise in nucleic acid to lipid (NA/L) ratio (*P* ≤ 0.039, Figure 2c–d). OVX/max had a higher NA/L ratio compared with the OVX/control group (*P* = 0.001, Figure 2c–d). There were no significant findings from protein to lipid (P/L) ratio (*P* ≥ 0.166, Figure 2c–d).

### 3.3 miRs displayed a greater portion of host sRNA cargo in EV than HDL particles

Since the nucleic acid cargo of EVs was the most affected by OVX and acute exercise, we continued by examining the sRNA species of EV and HDL particles. There was an average of 10.6 (8.4–15.8) million raw reads/sample in EV samples, whereas HDL samples had an average of 10.0 (6.4–14.3) million raw reads/sample. After adapter trimming, the average sRNA read count was 8.2 million in EVs and 8.4 million in HDL, of which miRs composed 144,000 in EVs and 103,000 in HDL.

**FIGURE 3:**
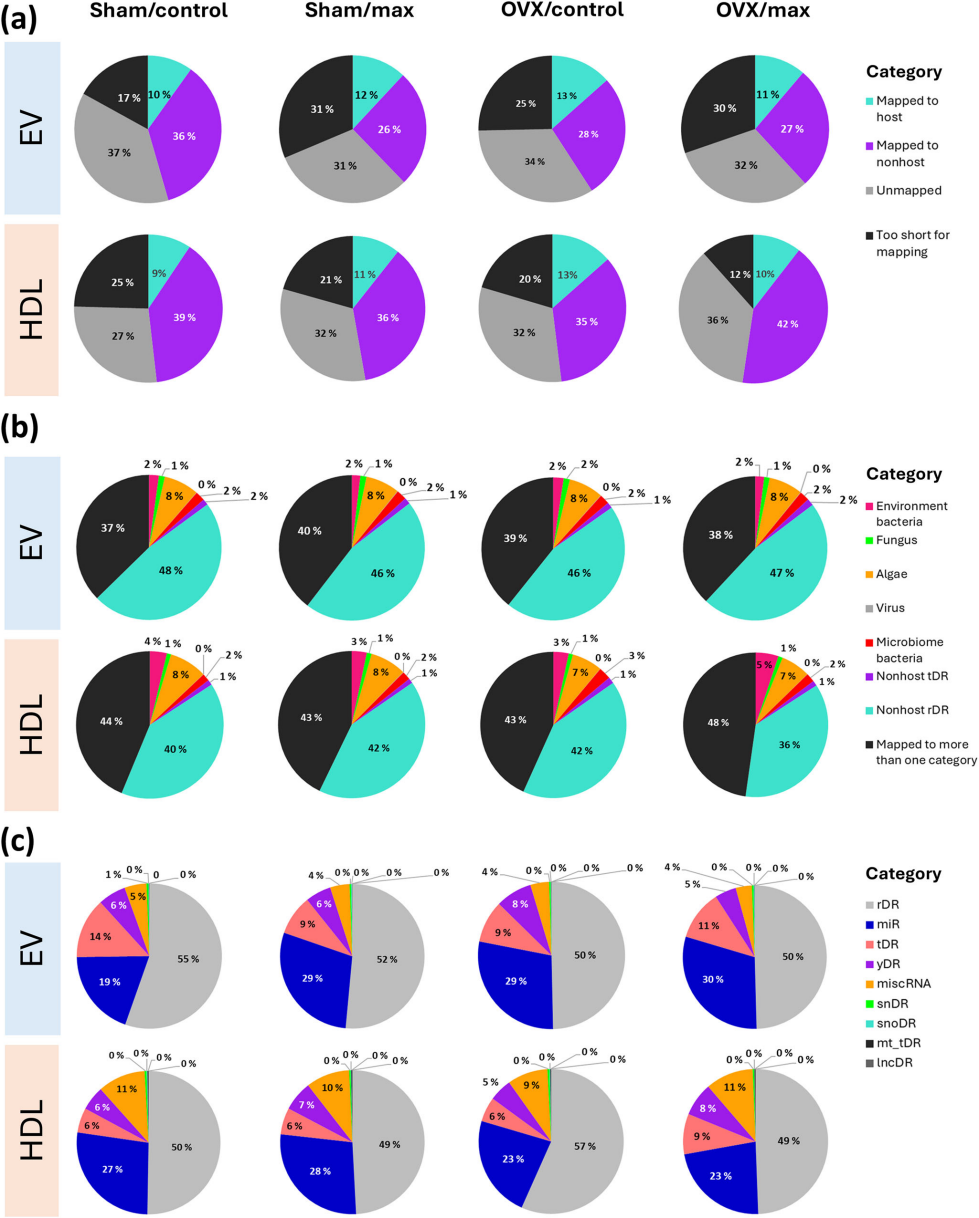
EV and HDL particle sRNA content in the study groups. (a) Majority of the mapped sRNAs of EV and HDL particles originated from non‐host. (b) Of the mapped sRNAs, most in EV and HDL particles were mapped to non‐host ribosomal RNA‐derived sRNAs (rDRs). (c) Of the host sRNAs, the majority were mapped to rDR in both EV and HDL particles. lncDR, long non‐coding (lncRNA)‐derived sRNA; miR, microRNA; miscRNA, miscellaneous RNA; mt_tDR, mitochondrial tDR; rDR, ribosomal RNA (rRNA)‐derived sRNA; snDR, small nuclear RNA (snRNA)‐derived sRNA; snoDR, small nucleolar RNA (snoRNA)‐derived sRNA; tDR, transfer RNA (tRNA)‐derived sRNA; yDR, yRNA.

We began our analysis by inspecting the sRNA origin in EV and HDL particles. In both particles, a major portion of the sRNAs were either unmapped or found to be too short for mapping (<16 nucleotides) (Figure 3a). Of the mapped sequences, a greater share was mapped to non‐host origin (26–36%), than to host origin (10–13%) (Figure 3a). The relative sRNA abundances were similar between groups, and EV and HDL particles also shared similarities (Figure 3b). The majority of EV non‐host sRNA sequences were mapped to rDR (46–48%), and to more than one category (37–40%) (Figure 3b). In HDL, most of non‐host sRNA sequences were mapped to the same categories as in EV particles, but more were mapped to more than one category than to rDR (43–48% and 36–42%, respectively, Figure 3b). Of the host sRNA sequences, the majority were mapped to rDR in both EV and HDL particles (50–55% and 49–57%, respectively, Figure 3c). In EVs, the second most common category was miRs, constituting 19–30% of all sRNA species. The abundance of miRs in EVs was lowest in the sham/control group (19%), whereas other groups did not portray differences in miR abundance (29% in sham/max and OVX/control, and 30% in OVX/max, Figure 3c). In HDL, miR abundance in host sequences was higher in the sham groups (27% and 28% in sham/control and sham/max, respectively), than in the OVX groups (23% in both OVX/control and OVX/max) (Figure 3c).

**FIGURE 4:**
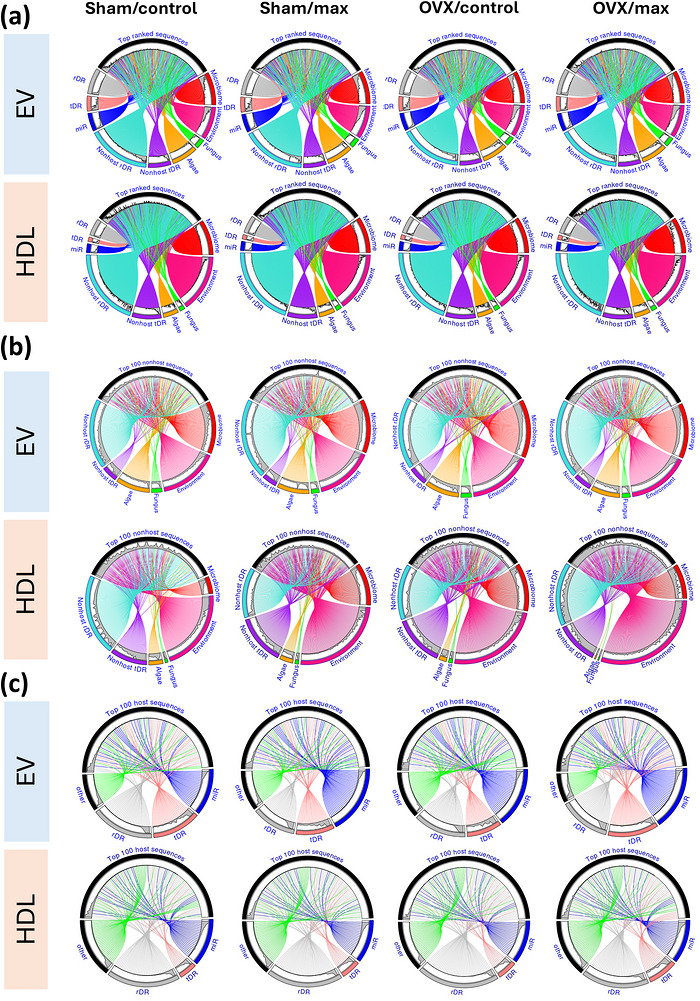
The top sRNA species of EV and HDL particles displayed minor differences. (a) Top ranked species in EV and HDL particles in each study group. (b) Top 100 ranked non‐host sRNA species in EV and HDL particles in each study group. (c) Top 100 host sRNA species in EV and HDL particles in each study group. miR, microRNA; rDR, ribosomal RNA (rRNA)‐derived sRNA; tDR, transfer RNA (tRNA)‐derived sRNA.

The top ranked sRNA sequences in EVs and HDL were similar between groups, with the major class being non‐host rDR (Figure 4a). In EVs, miRs constituted a larger proportion of sRNA classes compared to HDL particles (Figure 4a). Most of the top 100 non‐host sequences in EVs were mapped to rDR, while in HDL, the majority were environment‐derived sRNAs (Figure 4b). Most of the top 100 host sequences of EV sRNAs were mapped to miR, rDR and tDR. In HDL, the top 100 sRNA classes were the same as in EVs, with the distinction that tDRs constituted a smaller proportion than in EVs (Figure 4c).

### 3.4 The loss of ovarian hormones blunted the acute exercise‐induced miR response of EVs

**FIGURE 5:**
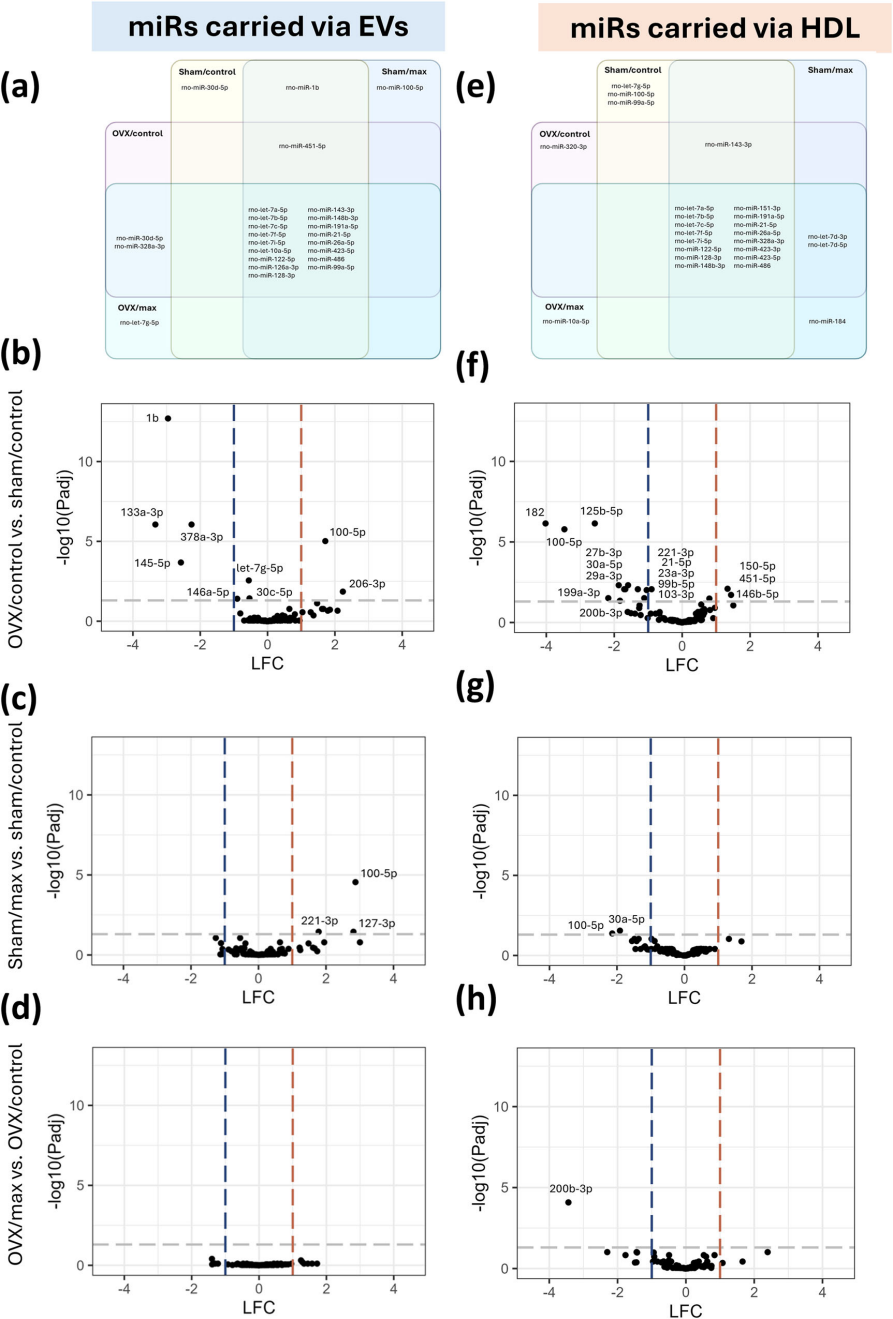
The miR cargo of EV and HDL particles responds to an acute bout of exercise differently. (a) Twenty most common miRs present in EVs (reads). (b–d) Volcano plots showing group comparisons of differential expression of miRs in EV particles. (e) Twenty most common miRs present in HDL (reads). (f–h) Volcano plots showing group comparisons of differential expression of miRs in HDL particles. LFC, log fold change; miR, microRNA; OVX, ovariectomy; Padj, adjusted *P*‐value.

We examined the 20 most common miRs in EV and HDL particles and observed 17 common miRs in EVs and 16 in HDL particles between the study groups (Figure 5a,e). The counts of all observed miRs are presented in Appendix Table A1.

We further examined the miR levels in EVs based on ovarian hormone status (OVX/control vs. sham/control), and the levels of nine miRs were significantly different between the groups (*P *< 0.050, Figures 5b,f, Appendix Table A2). Of these, miR‐1b, miR‐133‐2p, miR‐378a‐3p, miR‐30c‐5p, let‐7g‐5p and miR‐145‐5p were downregulated (*P *< 0.050, Figure 5b, Table A2), whereas miR‐100‐5p and miR‐206‐3p were upregulated (*P *< 0.050, Figure 5b, Table A2) in OVX. Furthermore, miR‐1b, let‐7g‐5p and miR‐100‐5p were among the 20 most common miRs in EVs, indicating a high biological relevance (Figure 5a, Table A1). When examining the effect of acute exercise in sham groups, there were three significantly upregulated miRs in EVs: miR‐100‐5p, miR‐127‐3p and miR‐221‐3p (*P *< 0.050, Figure 5c, Table A2). Of the significantly different miRs, miR‐1b, miR‐100‐5p and let‐7g‐5p were also among the 20 most common miRs (Figure 5a, Table A1). Excitingly, no differences between the OVX groups (OVX/max vs. OVX/control) were observed in EVs (*P *> 0.390, Figure 5d).

When examining the effect of ovarian hormones in HDL particles, there were 16 significantly different miRs: miR‐146b‐5p, miR‐150‐5p and miR‐451‐5p were upregulated (*P *< 0.050, Figure 5f, Appendix Table A3) and miR‐21‐5p, miR‐23a‐3p, miR‐27b‐3p, miR‐29a‐3p, miR‐30a‐5p, miR‐99b‐5p, miR‐100‐5p, miR‐103‐3p, miR‐125b‐5p, miR‐182, miR‐199a‐3p, miR‐200b‐3p and miR‐221‐3p downregulated (*P *< 0.050, Figure 5f, Table A3) in OVX. Of the significantly different miRs, miR‐21‐5p, 99b‐5p and 100‐5p were also among the 20 most common miRs (Figure 5e, Table A1). When examining the effect of acute exercise in sham groups, there were two significantly downregulated miRs in HDL particles: miR‐30a‐5p and miR‐100‐5p (*P *< 0.050, Figure 5g, Table A3). Interestingly, when comparing HDL miRs in OVX groups, miR‐200b‐3p was significantly downregulated (*P *< 0.001, Figure 5h, Table A3) in the exercised (max) group. However, miR‐200b‐3p was not among the 20 most common miRs indicating potentially minor physiological relevance (Table A1).

When studying miRs that respond differently to acute exercise based on the rat's ovarian hormone status (OVX/max vs. sham/max), the level of three miRs differed significantly in EVs: miR‐141‐3p showed upregulation (*P* = 0.038), and miR‐145‐5p and miR‐451‐5p were downregulated (*P* ≤ 0.038) (Appendix Figure A4). Of these, miR‐145‐5p was also found to be downregulated in EVs when comparing miRs based on the ovarian hormone status (OVX/control vs. sham/control, Figure 5b), indicating that this observed miR response is driven by the loss of ovarian hormones, independently from the effect of exercise. Only miR‐451‐5p was among the 20 most common miRs in EVs (Figure 5a), indicating that the other miRs may have smaller biological relevance.

In HDL particles, 10 miRs were found to be differentially expressed when comparing the effect of ovarian hormones in context of acute exercise (OVX/max vs. sham/max): let‐7f‐5p and miR‐10a‐5p were upregulated (*P* ≤ 0.031), whereas miRs 200b‐3p, 100‐5p, 199a‐3p, 27a‐3p, 155‐5p, 199a‐5p, 23a‐3p and let‐7g‐5p were downregulated (*P* ≤ 0.047) (Figure A4). Of these, miRs 200b‐3p, 100‐5p, 199a‐3p and 23a‐3p were also found to be downregulated in EVs when comparing miRs based on the ovarian hormone status (OVX/control vs. sham/control, Figure 5f). Of the differentially expressed miRs in this comparison, miRs let‐7f‐5p, 10a‐5p, 100‐5p and let‐7g‐5p were among the 20 most common miRs in EVs (Figure 5a), suggesting that they have a higher biological relevance.

**FIGURE A2:**
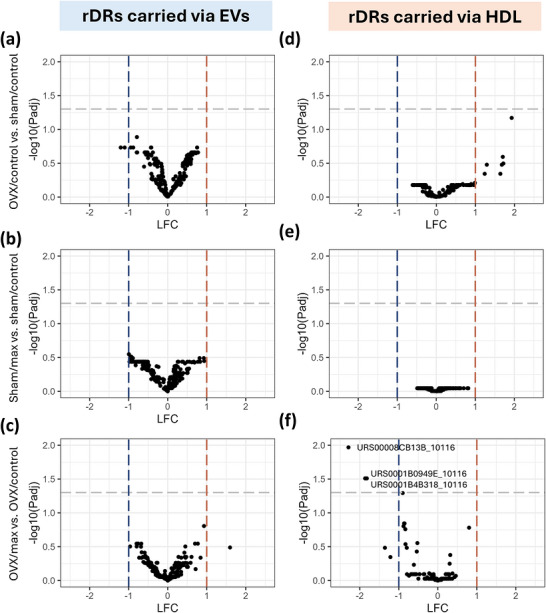
The rDR cargo of EV and HDL particles responds to acute bout of exercise differently. (a–c) Volcano plots showing group comparisons of differential expression of rDRs in EV particles. (d–f) Volcano plots showing group comparisons of differential expression of rDRs in HDL particles. OVX, ovariectomy; rDR, ribosomal RNA (rRNA)‐derived sRNA; LFC, log fold change.

**FIGURE A3:**
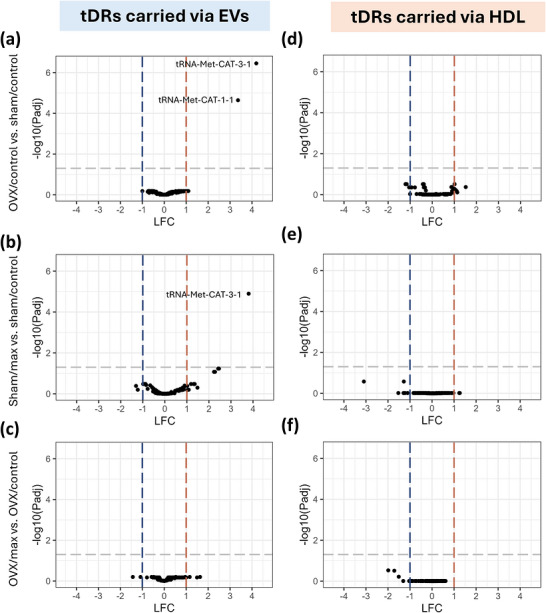
The tDR cargo of EV and HDL particles responds to acute bout of exercise differently. (a–c) Volcano plots showing group comparisons of differential expression of tDRs in EV particles. (d–f) Volcano plots showing group comparisons of differential expression of tDRs in HDL particles. OVX, ovariectomy; tDR, transfer RNA (tRNA)‐derived sRNA; LFC, log fold change.

**FIGURE A4:**
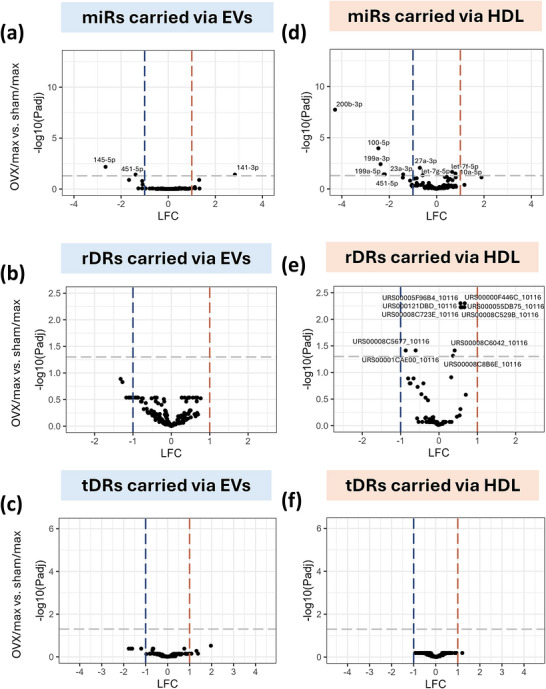
The miR and tDR cargoes of EV and HDL particles respond to acute bout of exercise differently in groups that differ in their ovarian hormone status. (a–c) Volcano plots showing group comparisons of differential expression of miRs, rDRs and tDRs, respectively, in EV particles. (d–f) Volcano plots showing group comparisons of differential expression of miRs, rDRs and tDRs, respectively, in HDL particles. LFC, log fold change; miR, microRNA; OVX, ovariectomy; rDR, ribosomal RNA (rRNA)‐derived sRNA; tDR, transfer‐RNA (tRNA)‐derived sRNA.

When examining the rDR and tDR species, we observed three significantly lower rDRs in HDL particles in OVX/max when comparing to OVX/control, all of which originated from small subunit ribosomal (SSU) rRNA (Figure A2, Appendix Table A4). There were no significant findings in rDRs carried via EVs (Figure A2, Appendix Table A5). No significant findings were observed in HDL in sham group comparisons, nor when comparing control groups (Figure A2, Table A4). However, 11 rDRs were differentially expressed in HDL when comparing OVX/max to sham/max: URS00000F446C_10116, URS00005F96B4_10116, URS0000121DBD_10116, URS000055DB75_10116, URS00008C45F1_10116, URS00008C529B_10116, URS00008C723E_10116, URS00008C6042_10116 and URS00008C8B6E_10116 were upregulated (*P* ≤ 0.048), whereas URS00001CAE00_10116 and URS00008C5677_10116 were downregulated (*P* ≤ 0.039) (Figure A4, Table A4). In tDRs, there were two significantly higher species in EVs when comparing control groups of OVX and sham: tRNA‐Met‐CAT‐3‐1 and tRNA‐Met‐CAT‐1‐1 (*P* ≤ 0.000). Only tRNA‐Met‐CAT‐3‐1 was significantly higher in sham/max versus sham/control (*P* < 0.000) (Figure A3, Appendix Table A6). There were no significant differences in tDRs between the OVX groups or max groups in EVs or in the tDRs carried via HDL particles between any of the studied groups (Figures A3 and A4, Appendix Table A7).

### 3.5 The exercise‐responsive miR cargo of EVs and HDL may affect energy metabolism in skeletal muscle through target proteins

To study the ovarian hormone‐responsive miRs’ functional relevance, a miR‐mRNA pathway analysis was carried out with miRPath v3.0 (Vlachos et al. 2015). The analysis was performed for all miRs found to be differentially expressed between OVX/control and sham/control, that is for nine EV‐carried, and 16 HDL‐carried miRs (Figure 5b,f, Table A2). Central pathways to energy production were analysed further, and target proteins of differentially expressed miRs were examined from these pathways.

**FIGURE 6:**
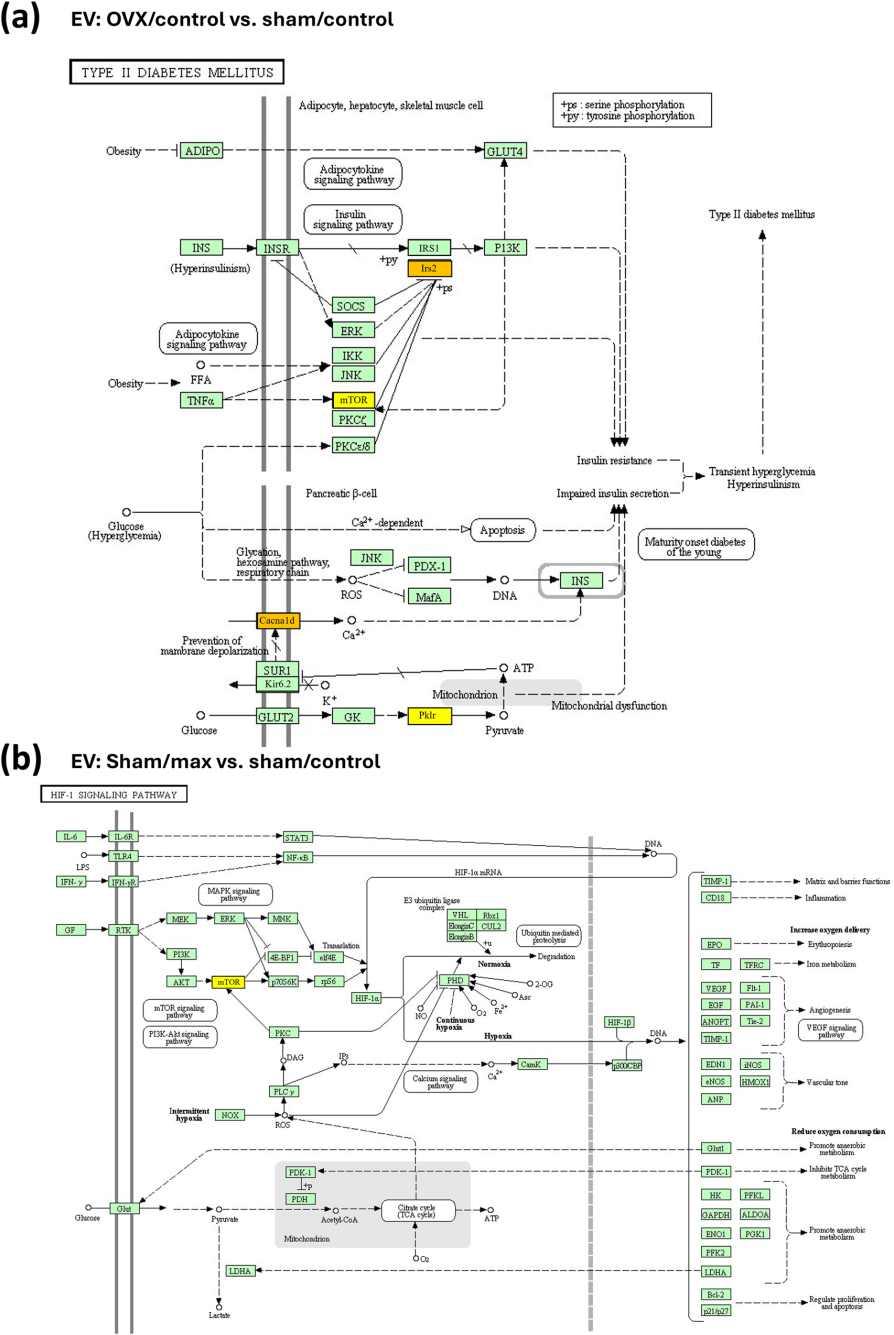
miR target pathways (miRPath v3.0), illustrating signalling pathways regulated via differentially expressed miRs in EV particles. (a) Visualization of type 2 diabetes signalling pathway and target proteins regulated by significantly different miRs carried by EV particles. (b) Visualization of HIF‐1 signalling pathway and target proteins regulated by significantly different miRs carried by EV particles. Yellow, protein regulated by one miR; orange, protein regulated by more than one miR. CACNA1D, calcium voltage‐gated channel subunit α1 D; IRS2, insulin receptor substrate 2; mTOR, mammalian target of rapamycin; OVX, ovariectomy; PKLR, pyruvate kinase L/R.

Analysis of EV miRs, using a Kyoto Encyclopedia of Genes and Genomes (KEGG) analysis with pathways union option showed two pathways to be significantly regulated by three of the studied EV‐carried miRs, involving type 2 diabetes pathway (Figure 6a, Appendix Table A8). Type 2 diabetes signalling pathway regulation by the three miRs was mediated through the following target proteins: mTOR, PKLR, IRS2 and calcium voltage‐gated channel subunit α1 D (CACNA1D) (Figure 6a). Similar analysis of ovarian hormone‐responsive HDL miRs revealed seven pathways to be targeted by four of the studied miRs (Appendix Table A9).

When studying the exercise‐responsive EV miRs (sham/max vs. sham/control), miRPath v3.0 KEGG pathway analysis with pathways union option showed 10 pathways, which were regulated by two of the miRs (Table A8). Of these, hypoxia inducible factor 1 (HIF‐1) pathway, which plays a role in vascularization, is targeted by miR‐100‐5p, through its target protein mTOR (Figure 6b). When examining the two exercise‐responsive HDL miRs, we observed 30 significant pathways, all of which were exclusively regulated by miR‐100‐5p, including type 2 diabetes pathway (Table A9).

When studying the exercise‐responsive HDL miRs in groups differing in their ovarian hormone status (OVX/max vs. sham/max), miRPath v3.0 KEGG pathway analysis with pathways union option showed 10 pathways, which were regulated by 10 of the differentially expressed miRs (Figure A4), including fatty acid degradation pathway (Table A9). There were no differentially expressed miRs in EVs between OVX/max and sham/max (Figure A4).

**FIGURE 7:**
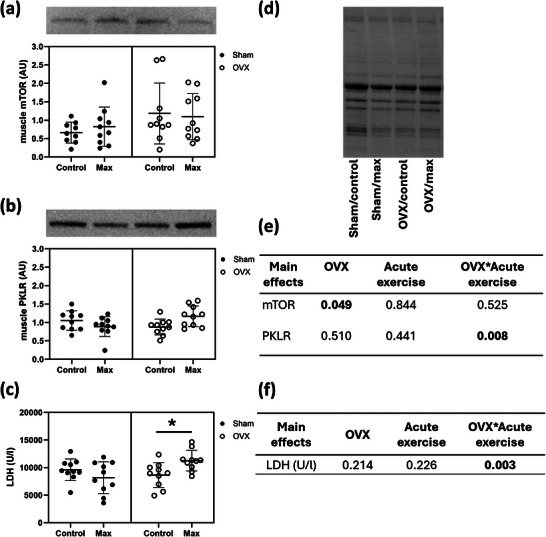
Loss of ovarian hormones and acute bout of exercise affect miR target protein levels in skeletal muscle (data reported as means, and error bars representing standard deviations where applicable). (a) Western blot analysis of mTOR level in muscle. (b) Western blot analysis of muscle PKLR. (c) Enzyme activity assay of LDH in muscle. (d) Western blot image of total protein, against which the results were normalized. (e) Main effects of OVX, acute exercise and their interaction on mTOR and PKLR levels in muscle (*P*‐values). (f) Main effects of OVX, acute exercise and their interaction on LDH activity (*P*‐values). LDH, lactate dehydrogenase; mTOR, mammalian target of rapamycin; OVX, ovariectomy; PKLR, pyruvate kinase L/R.

**FIGURE A5:**
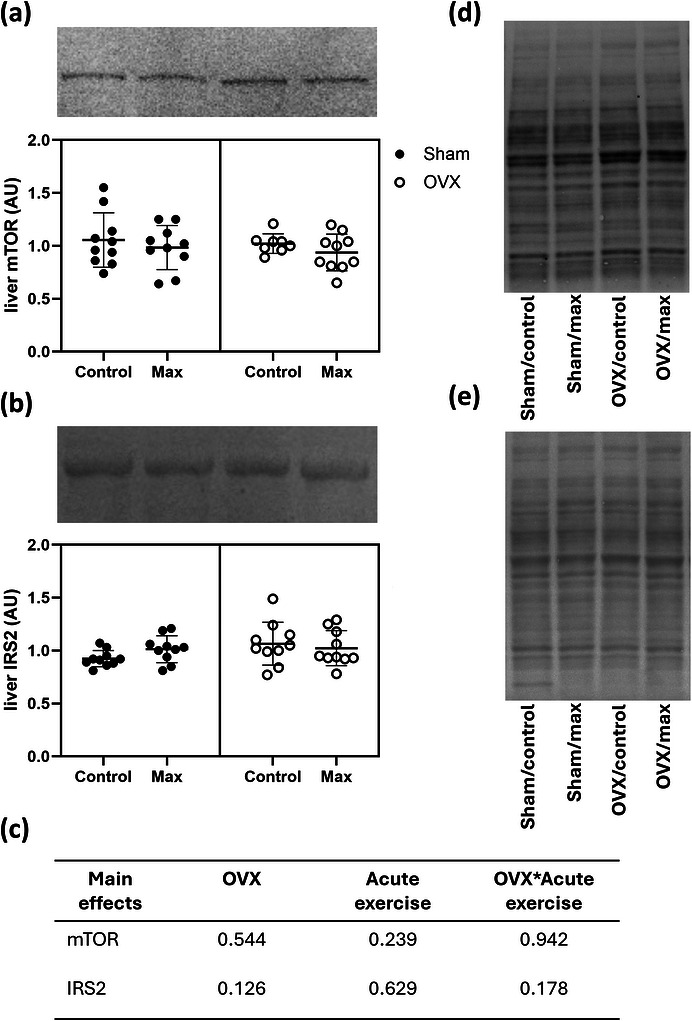
Loss of ovarian hormones and acute bout of exercise did not affect the miR target protein levels in liver. (a) Western blot analysis of mTOR level in liver. (b) Western blot analysis of muscle IRS2 level in liver. (c) Main effects of OVX, acute exercise and their interaction on mTOR and IRS2 levels in liver. (d–e) Corresponding western blot images of total protein, against which the results were normalized. OVX, ovariectomy; mTOR, mammalian target of rapamycin; IRS2, insulin receptor substrate 2.

To elucidate the possible functional relevance of the differentially expressed miRs in liver and muscle tissue, western blot analysis was carried out for the most relevant target proteins from miRPath v3.0 analysis (Figure 6). Unfortunately, antibodies for CACNA1D proved to be defective, and therefore western blot was carried out with mTOR (both muscle and liver), PKLR (only muscle) and IRS2 (only liver) (Figure 7a,b, Appendix Figure A5). The differentially expressed miRs and target protein expression in liver had no association (*P* ≥ 0.126, Figure A5), but changes in the target proteins were observed in skeletal muscle tissue (Figure 7a,b,d,e). Muscle mTOR expression was lower in sham than in OVX (*P* = 0.049, Figure 7a,e). There was an interaction of OVX and acute exercise in muscle PKLR expression (*P* = 0.008, Figure 7b,e).

Since PKLR is an integral part of glycolysis, as it catalyses the conversion of phosphoenolpyruvate to pyruvate in the last step of glycolysis, we hypothesized that the change in PKLR induced by miR signalling changes would be reflected on the lactate dehydrogenase (LDH) enzyme activity, which is the next step in anaerobic glycolysis. LDH catalyses the production of lactate from pyruvate, and its activity is a commonly used marker of the level of anaerobic energy production. The loss of ovarian hormones resulted in higher LDH activity in response to an acute bout of exercise (*P* = 0.003) (Figure 7c,f).

## 4 DISCUSSION

In this study, we examined the effect of loss of ovarian hormones and acute exercise in the sRNA cargo of EV and HDL particles in female rats. In rats, menopause is mimicked by ovariectomy (OVX). OVX leads to a dramatic drop in ovarian hormones, including E2 and progesterone, with E2 being the main contributor to negative effects on health similarly as in post‐menopausal women (Karvinen et al. 2023). We show for the first time that loss of ovarian hormones affects specifically the nucleic acid cargo of circulating EVs. We have demonstrated before that acute exercise‐induced miR‐response in EV and HDL particles is absent in postmenopausal women with low ovarian hormone levels (Karvinen et al. 2023), which was confirmed here in a rat model. We further demonstrate that the oestrogen‐responsive miRs coordinate the glycolytic pathway. Finally, we show that the loss of ovarian hormones leads to higher anaerobic energy production during an acute bout of exercise, suggesting an inferior ability to sustain aerobic energy production during exercise.

Loss of ovarian hormones increased body fat mass and reduced maximal running capacity as shown previously in our larger study cohort (Lee et al. 2024). Several studies have demonstrated that the loss of ovarian function in women and female rats is linked to weight gain, an increase in adipose tissue mass and deterioration in metabolic health (Asarian & Geary, 2002; Juppi et al. 2022; Lee et al. 2024). Furthermore, loss of ovarian hormones has been associated with decreased physical activity levels in rodents (Chen et al. 2014; Park et al. 2016), and similar findings have been associated with the menopause in women (Laakkonen et al. 2017; Pettee Gabriel et al. 2015). These findings together with our current results indicate inter‐species similarity in responses to loss of ovarian hormones.

We show for the first time that loss of ovarian hormones changes specifically the nucleic acid cargo of EVs. A previous study by Sahu et al. (2021) observed that ageing changed specifically the nucleic acid cargo of circulating EVs in a mouse model. To our knowledge, we demonstrate for the first time that also loss of ovarian hormones leads to similar effects. Taken together, these findings indicate that ageing‐related alterations specifically impact the EV RNA cargo. Accordingly, we continued with a detailed analysis of the sRNA species carried via EV and HDL particles.

In the present study the relative abundance of miRs was between 19% and 30% of host sRNAs and it represented the first or second most abundant sRNA species of the top 100 host sRNAs. Further examination revealed that loss of ovarian hormones affected miRs associated with type 2 diabetes signalling pathways in circulating EVs. When comparing sham and OVX groups, we found nine miRs to be differentially expressed in EVs, and 16 in HDL particles. Of the EV miRs, let‐7g‐5p, miR‐100‐5p and miR‐145‐5p were associated to genes related to type 2 diabetes. In this pathway, let‐7g‐5p and miR‐145‐5p had targeted proteins CACNA1D and IRS2, while let‐7g‐5p targeted also PKLR. The target protein of miR‐100‐5p in this pathway was mTOR. Of these, let‐7g‐5p and miR‐100‐5p were also among the 20 most common miRs, highlighting their physiological relevance. In HDL particles, the differentially expressed miRs 100‐5p and 99b‐5p were associated with the phosphoinositide 3‐kinase (PI3K)/protein kinase B (PKB, otherwise known as AKT) signalling pathway. Both of the miRs had fibroblast growth factor receptor 3 (FGFR3), and miR‐100‐5p had also mTOR, as a target protein. The PI3K/AKT pathway is mediated by insulin, affecting energy metabolism in insulin‐sensitive tissues (Cao et al. 2023). In obesity, both up‐ and downregulation of the PI3K/AKT pathway have been found to be beneficial in a context‐dependent manner (Savova et al. 2023). Our findings highlight the significant impact of loss of ovarian hormone on the nucleic acid cargo, particularly in miRs involved in type 2 diabetes and insulin signalling pathways, underscoring the complex interplay between hormonal changes and metabolic regulation.

Our previous research has shown that in postmenopausal women, low systemic oestrogen levels reduce the miR response to exercise in both EV and HDL particles (Karvinen et al. 2023). Our present results confirm that the loss of ovarian hormones blunts the exercise‐induced miR response of EVs also in female rats. While in the sham groups there were three exercise‐responsive miRs, none were observed in the OVX group. Interestingly, even though the OVX group had lower maximal running capacity in the POST test compared to sham, in the END measurement, where the running test was performed only once, there was no difference between the groups. This observation suggests that the difference between the systemic response to exercise in OVX rats is not attributable to a reduced running stimulus but rather represents a physiological change.

Of the exercise‐responsive EV miRs in sham groups, miR‐100‐5p was upregulated and found to be associated with hypoxia‐inducible factor‐1 (HIF‐1) signalling through mTOR. However, we did not observe a difference in the mTOR level in skeletal muscle in response to exercise. HIF‐1 target genes act in skeletal muscle in oxygen transport enhancement through, for example, activation of angiogenesis and glycolysis (Lindholm & Rundqvist, 2015). The upregulation of miRs targeting HIF‐1 in the sham/max group may indicate attenuated HIF‐1 response to acute exercise stimulus.

When studying EV miRs, that respond differently to acute exercise based on the rats’ ovarian hormone status (OVX/max vs. sham/max), miR‐145‐5p was downregulated, as in the comparison based solely on the ovarian hormone status (OVX/control vs. sham/control). This suggests that the response to acute exercise of this specific miR is driven by the loss of ovarian hormones, independently from the effect of exercise. However, the differential expression of upregulated miR‐141‐3p and downregulated miR‐451‐5p is not explained by the ovarian hormones alone, since these miRs were not found to differ in control groups. Only miR‐451‐5p were among the 20 most common miRs in EVs (Figure 5a), indicating that the other miRs may have smaller biological relevance. In humans, the level of plasma miR‐451a (that is 451‐5p; Yang et al. 2010) has been shown to be lower in both endurance and resistance athletes compared with sedentary controls (Fernandez‐Sanjurjo et al. 2024), yet upregulated after an acute, fatiguing bout of endurance exercise (D'Souza et al. 2018; Lai et al. 2023; Nair et al. 2020). While miR‐451a has been shown to have a key role in mTOR signalling (Minna et al. 2016), in this study we observed no changes in the mTOR protein level in skeletal muscle or liver tissue in response to acute exercise. The role of miR‐451‐5p in the context of acute exercise warrants further research.

When studying HDL miRs that respond differently to acute exercise based on the rats’ ovarian hormone status (OVX/max vs. sham/max), miRs 200b‐3p, 100‐5p, 199a‐3p and 23a‐3p were also found to be downregulated when comparing HDL miRs based on the ovarian hormone status (OVX/control vs. sham/control). Therefore, it is likely that the observed difference in these specific miRs is caused by the loss of ovarian hormones rather than acute exercise. Of the unique, differentially expressed exercise‐responsive miRs in this comparison, upregulated miRs let‐7f‐5p, 10a‐5p, and downregulated 100‐5p and let‐7g‐5p were among the 20 most common miRs in EVs, suggesting that they have a higher biological relevance. In urinary EVs, let‐7f‐5p has been shown to be upregulated in response to menopause (Maham et al. 2025), similarly as what was seen in our study design, but to our knowledge, its role in exercise has not been studied. In rats with normal ovarian function, the level of HDL miR‐100‐5p was downregulated in response to acute exercise (sham/max vs. sham/control), suggesting that miR‐100‐5p is exercise‐responsive regardless of the influence of ovarian hormones. The potential association of miRs 10a‐5p and let‐7g‐5p to either exercise or ovarian hormones has not been addressed by previous literature, warranting more research in their possible contributions to exercise responses.

Excitingly, there was an interaction of OVX and acute exercise on the level of PKLR in skeletal muscle. It seems that in response to exercise, the PKLR level in muscle decreases in the sham group, whereas in OVX the PKLR level increases. However, in addition to the main effect of OVX and acute exercise interaction, no significant findings between the control and corresponding max groups were observed. PKLR takes part in energy production through glycolysis (Israelsen & Vander Heiden, 2015). PKLR catalyses the second to last step of anaerobic glycolysis, which converts phosphoenolpyruvate to pyruvate in ATP production. Hence, elevated PKLR levels may indicate increased glycolytic activity, likely as a response to higher energy demands in the muscle. LDH in turn catalyses the last step of anaerobic glycolysis, converting pyruvate to lactate. In our study, the OVX group exhibited higher LDH activity in the max group, further indicating that the glycolytic flux is higher in response to acute exercise with low ovarian hormone status.

Of the ovarian hormones, E2 has been proven to act as key regulator of energy homeostasis affecting several tissues, including skeletal muscle (Mauvais‐Jarvis et al. 2013). E2 promotes lipid oxidation in skeletal muscle in vivo and in vitro (Garrido et al. 2014; Hamadeh et al. 2005), while OVX may lead to reduced oxidative capacity due to impaired mitochondrial function (Hu et al. 2024; Torres et al. 2018). We found that muscle mTOR was higher in OVX than sham. mTOR is known for its diverse roles in metabolism regulation (Saxton & Sabatini, 2017; Shirai et al. 2021). mTOR signalling regulates LDH as a downstream target by promoting its expression and activity (Manerba et al. 2018). To support this observation, we also discovered a higher LDH activity in the OVX group in response to acute exercise, indicating a higher level of anaerobic energy production. Our results are in line with previous literature, yet to our knowledge, our study is the first to show that loss of ovarian hormones leads to higher anaerobic energy production in response to an acute bout of exercise. Our results suggest that OVX rats are less effective at sustaining aerobic energy production during an acute bout of exercise.

We observed one exercise‐responsive miR in the HDL particles in the OVX group, while there were two such miRs in the sham group. In the OVX group, miR‐200b‐3p was downregulated in response to exercise (OVX/max vs. OVX/control). Patients with type 2 diabetes have been shown to have less circulating miR‐200b in plasma than healthy controls (Dantas da Costa e Silva et al. 2019). Also, coronary artery disease patients have been shown to display downregulation of miR‐200b‐3p after an acute bout of exercise (Mayr et al. 2019). In cultured alveolar cells, miR‐200b‐3p has been shown to be capable of decreasing senescence markers and restoring regenerative potential (Moimas et al. 2019). Taken together, these findings suggest that the downregulation of miR‐200b‐3p may be linked to negative cardiovascular factors. Our finding of the downregulation of this specific miR in HDL particles in the OVX group may indicate that miR‐200b‐3p is exercise‐responsive and is affected by the loss of ovarian hormones, possibly providing a link between unfavourable cardiovascular changes associated with the loss of ovarian hormones.

In EV and HDL particles, the relative abundance of rDR was between 49% and 57% and it represented the first or second most abundant sRNA species of the top 100 host sRNAs. Interestingly, we found significant differences in rDR species only in the HDL particles when comparing the OVX groups (max vs. control), suggesting that the loss of ovarian hormones together with acute exercise modulates the rDR cargo of HDL, whereas acute exercise or OVX alone does not cause changes. All of these rDRs originated from SSU rRNA. SSU rRNA is one of the two main RNA components of a ribosome, also known as 18S rRNA. SSU rRNA plays a crucial role in the translation of genetic information into proteins. In our previous study the majority of the exercise‐responsive rDR species in HDL particles belonged to mitochondrial ribosomal subunits (Karvinen et al. 2023). While mitochondrial peptides are now known to regulate metabolism (Kim et al. 2017), the potential role of SSU rRNA fragments in signalling remains uncovered.

In EV and HDL particles, the relative abundance of tDR was between 6% and 14% and it represented the third most abundant sRNA species of the top 100 host sRNAs. In EVs, tDR derived from tRNA‐Met‐CAT‐3‐1 was higher both in OVX rats compared with sham and in sham/max compared with sham/control group. In addition, tDR derived from tRNA‐Met‐CAT‐1‐1 was upregulated in OVX rats compared with sham. However, no effect of acute exercise in EV tDRs was seen when comparing OVX/max to OVX/control. tRNA fragments, while still under thorough investigation, have been found to play significant roles in systemic signalling, impacting various biological processes (Kaimwal et al. 2025; Torres & Martí, 2021). Their currently known biological roles include regulating gene expression by inhibition of translation (Lee et al. 2009; Li et al. 2012). Although tDRs may have similar regulatory functions to miRs, their role as systemic signalling molecules remains unclear.

To conclude, this study highlights the role of ovarian hormones in regulating miR signalling in response to acute exercise. Accordingly, the miR cargo of circulating EVs may affect the protein expression of the target tissues and influence cellular metabolism. Our findings emphasize the importance of ovarian hormones in the acute response to exercise and their potential role in facilitating the health benefits associated with exercise. If these findings were replicated in women, it could shed light on, for example, exercise recommendations in post‐menopausal women – if the exercise‐response is disrupted, a larger volume or more intense exercise might be needed to reach similar benefits as in pre‐menopausal women.

### 4.1 Strengths and limitations

The present study was designed to investigate the effects of OVX and acute bout of exercise on systemic signalling. The first key strength of the current study is that the animals were fully grown adults (∼7 months old) before undergoing surgery, ensuring a more robust model of menopause. Second, to ensure a robust difference in the ovarian hormone levels between OVX and sham groups, the rats in the sham group were euthanized at the time of pro‐oestrus. The third strength of the study is the implementation of allocating animals into the four study groups matched for body weight and maximal running capacity. Fourth, we utilized an isolation protocol validated by us enabling the separation of EV and HDL fractions before RNA isolation. Our study also has certain limitations. Although we did not measure circulating oestrogen levels due to the need to allocate all plasma for EV isolation, uterine mass is a well‐established and widely accepted indicator of long‐term oestrogen deficiency (see e.g., Erben et al. 2004). Therefore, it serves as a reliable surrogate for assessing ovarian hormone loss in long‐term studies. Due to the low protein content of the EV samples, we were not able to carry out the protein analysis via western blot and hence utilized dot blot analysis when necessary. Nevertheless, total protein, dot blot and EM analyses confirmed that the EV and HDL particles were enriched in separate fractions. We were not able to detect all the target proteins in both muscle and liver tissues due to defectiveness or tissue specificity of the antibodies. While functional miR analyses in cell culture models were beyond the scope of the present study, such experiments would serve as a valuable follow‐up to further explore the underlying mechanisms. Also, due to the limited sample volume we were not able to assess the number of EV or HDL particles in the samples. Therefore, the contribution of altered miR loading versus differences in particle number to the observed differentially expressed miRs and LDH activity remains uncovered. Nevertheless, we are confident that the results are relevant regardless of whether the observed differences between the groups are partly due to the increased number of particles, as only a few specific miRs showed a clear response to exercise stimulus in the sham group, supporting highly coordinated regulatory mechanisms for miR packaging. Due to the strong resonance Raman effect of carotene molecules, which overpowers the Raman spectra of lipids and proteins, the P/L and NA/L ratios could not be determined from the HDL sample with our measurement protocol. Also, we were not able to trace the EV and HDL particles to determine their target tissues, yet based on previous literature, we are confident that liver and muscle are affected by systemic signalling (Vechetti et al. 2021; Whitham et al. 2018).

## AUTHOR CONTRIBUTIONS

Veera Puumalainen: Methodology (western blot, miR‐pathway); analysis; writing—original draft. Anni Maja: Methodology (EV analysis); analysis; writing (supportive). Tia‐Marje Korhonen: Analysis; writing (review and editing). Tuuli A. Nissinen: methodology (animal study); writing (review and editing). Eero Hulkko: Methodology (Raman spectroscopy); analysis; writing (review and editing). Janne A. Ihalainen: Resources (Raman spectroscopy); methodology; writing (review and editing). Maarit Lehti: Methodology; supervision (supportive); writing (review and editing). Sira Karvinen: Conceptualization (lead); resources (lead); supervision (lead); writing—review and editing (lead). All authors have read and approved the final version of this manuscript and agree to be accountable for all aspects of the work in ensuring that questions related to the accuracy or integrity of any part of the work are appropriately investigated and resolved. All persons designated as authors qualify for authorship, and all those who qualify for authorship are listed.

## CONFLICT OF INTEREST

None declared.

## GENERATIVE AI STATEMENT

No generative AI tools have been used in the preparation of this article.

## Data Availability

The results obtained are presented in the manuscript. When applicable, a link for the data source is presented.

## 1

Ovarian hormone loss modifies the nucleic acid cargo of circulating extracellular vesicles and skeletal muscle metabolism after acute exercise in rats.

**TABLE A1:** miR counts in EV and HDL particles in the study groups.

		EV				HDL			
Number	miR	Sham/control	Sham/max	OVX/control	OVX/max	Sham/control	Sham/max	OVX/control	OVX/max
1	let‐7a‐5p	33610	54906	58282	57313	24073	27751	29935	41685
2	let‐7b‐5p	45021	59732	64835	52030	31015	43941	39991	62414
3	let‐7c‐2‐3p	71	145	182	190				
4	let‐7c‐5p	25250	43464	40962	36856	21470	30590	25387	39516
5	let‐7d‐3p	6476	10640	10935	9486	8172	11896	9115	13803
6	let‐7d‐5p	6583	10873	11244	10010	7876	8678	7439	9487
7	let‐7e‐5p	1245	1898	1692	1708	831	849	862	1201
8	let‐7f‐5p	49439	77149	77945	77001	27636	35066	38822	60539
9	let‐7g‐5p	9834	10823	10587	15947	7211	6879	5349	4563
10	let‐7i‐5p	110030	152200	170430	145955	94198	114086	95914	139715
11	miR‐100‐5p	1823	14890	8578	12454	5731	3427	793	564
12	miR‐103‐3p	773	1138	1098	1449	993	800	607	740
13	miR‐101a‐3p	256	357	554	596				
14	miR‐106b‐3p	276	540	511	449	217	397	339	312
15	miR‐101b‐3p	73	87	148	111				
16	miR‐10a‐5p	18255	30909	23445	31622	4308	4443	5412	8885
17	miR‐122‐5p	24484	67542	56950	20219	46599	26837	38543	16884
18	miR‐125a‐5p	3486	5752	4836	7268	1584	1313	987	1904
19	miR‐125b‐5p	448	872	636	943	1618	1037	261	450
20	miR‐126a‐3p	20634	28338	24071	31638	4711	6256	5407	7744
21	miR‐127‐3p	113	282	420	534	404	290	145	185
22	miR‐128‐3p	51775	82806	85128	71748	88676	114676	86417	108088
23	miR‐139‐5p	624	1229	852	846	211	317	356	441
24	miR‐1‐3p	845	1352	1280	1421	1088	644	598	2162
25	miR‐132‐3p	114	144	258	279				
26	miR‐142‐3p	596	583	816	1183	317	414	288	462
27	miR‐133a‐3p	299	198	56	341				
28	miR‐143‐3p	27269	26926	36466	20871	16519	10529	10151	6960
29	miR‐146a‐5p	1786	1601	1627	2593	486	718	600	773
30	miR‐146b‐5p	2727	4258	6622	15744	1075	1218	1687	2374
31	miR‐141‐3p	120	101	134	320				
32	miR‐148a‐3p	754	1117	1233	821	300	369	329	287
33	miR‐148a‐5p	190	205	253	295	171	202	262	257
34	miR‐142‐5p	98	254	408	362				
35	miR‐148b‐3p	231318	364421	413206	330739	220010	296548	265971	314655
36	miR‐150‐5p	803	1342	1403	2412	554	714	570	303
37	miR‐145‐5p	327	197	123	37				
38	miR‐151‐3p	6648	11039	10861	9554	7764	10150	8099	9850
39	miR‐152‐3p	142	161	208	216	174	116	76	79
40	miR‐155‐5p	246	870	1615	4677	185	202	207	40
41	miR‐16‐5p	427	378	398	593	192	177	147	157
42	miR‐17‐5p	352	585	580	773	460	383	506	349
43	miR‐181a‐5p	824	1473	1427	2241	1331	1461	927	1110
44	miR‐181b‐5p	291	504	432	414	278	295	270	457
45	miR‐181d‐5p	466	891	707	670	952	827	967	1075
46	miR‐182	89	367	325	471	339	244	36	138
47	miR‐184	3771	8001	5312	5505	1653	8846	1797	8447
48	miR‐185‐5p	248	335	332	490	443	335	383	434
49	miR‐186‐5p	522	877	1235	1353	336	485	458	677
50	miR‐191a‐3p	317	399	544	360	562	789	602	792
51	miR‐191a‐5p	25681	39373	39575	40445	49910	50594	50778	52992
52	miR‐192‐5p	212	355	438	334	293	201	1203	127
53	miR‐199a‐3p	583	497	486	438	1199	1017	200	164
54	miR‐183‐5p	87	231	451	720				
55	miR‐199a‐5p	190	302	222	636	369	264	98	44
56	miR‐1b	22681	10881	4690	10326	4739	2267	3014	3098
57	miR‐200b‐3p	628	827	1528	1995	2535	1376	528	72
58	miR‐206‐3p	200	1107	1048	1385	1093	574	355	1635
59	miR‐20a‐5p	481	576	824	1061	417	450	469	471
60	miR‐210‐3p	205	243	294	411	308	299	184	213
61	miR‐21‐5p	32896	55624	69432	101693	31153	32026	21897	27841
62	miR‐221‐3p	221	735	908	1700	757	600	320	251
63	miR‐223‐5p	2424	3092	3539	4661	1243	1580	1484	2500
64	miR‐22‐3p	1107	1876	1873	1994	996	1103	1509	1268
65	miR‐200a‐3p	271	276	241	263				
66	miR‐22‐5p	153	358	474	198	164	172	189	215
67	miR‐23a‐3p	172	107	214	248	294	250	115	96
68	miR‐203a‐3p	85	407	213	230				
69	miR‐24‐2‐5p	336	416	406	403	341	364	361	357
70	miR‐204‐3p	84	72	308	177				
71	miR‐24‐3p	4838	6906	7251	7574	4868	5700	4765	5785
72	miR‐25‐3p	2618	3845	4519	5839	2246	2751	2503	2643
73	miR‐26a‐5p	67717	120248	120772	123020	36106	46237	49017	67410
74	miR‐26b‐5p	393	631	725	744	319	348	315	279
75	miR‐27a‐3p	2330	4123	5608	7006	2547	2942	2140	1967
76	miR‐218a‐5p	79	286	244	69				
77	miR‐27a‐5p	219	361	455	595	148	169	195	276
78	miR‐27b‐3p	2175	2217	2563	2402	4463	2696	1144	892
79	miR‐28‐3p	419	686	991	1197	617	568	642	658
80	miR‐29a‐3p	357	372	482	693	377	301	215	167
81	miR‐30a‐3p	252	482	570	446	260	232	193	224
82	miR‐30a‐5p	3164	3078	3763	3083	1499	931	817	1078
83	miR‐30c‐5p	3279	3642	7399	16656	2197	2280	2563	2543
84	miR‐30d‐5p	11005	16220	18384	19473	5986	5915	6505	7952
85	miR‐30e‐3p	562	1087	2771	5732	393	458	586	638
86	miR‐30e‐5p	1400	2365	4135	7303	447	546	836	995
87	miR‐320‐3p	5480	7926	8065	7961	6219	6209	7617	6194
88	miR‐328a‐3p	8198	12513	11703	10188	20721	19495	17312	19409
89	miR‐330‐3p	815	1246	1312	1002	1025	1109	1028	1550
90	miR‐28‐5p	70	126	195	150				
91	miR‐339‐3p	120	125	259	314	201	274	228	308
92	miR‐339‐5p	104	284	263	422	506	495	365	512
93	miR‐340‐3p	550	852	1066	1305	269	315	251	418
94	miR‐340‐5p	429	501	790	1121	192	168	109	284
95	miR‐342‐5p	1231	1852	2135	1660	977	1336	914	1981
96	miR‐31a‐5p	31	114	148	142				
97	miR‐361‐3p	562	730	866	890	618	534	462	1018
98	miR‐378a‐3p	997	514	341	362	330	234	235	210
99	miR‐423‐3p	5051	8541	10418	13375	9351	10614	9454	10269
100	miR‐423‐5p	28116	39544	40552	31661	28736	36733	32985	43251
101	miR‐425‐5p	3405	5806	4869	4932	7046	6651	5938	8300
102	miR‐451‐5p	11478	18184	10075	6031	1985	2730	4908	4330
103	miR‐484	251	562	255	909	577	559	409	719
104	miR‐486	20731	36551	25560	27950	9529	18489	13482	20936
105	miR‐34c‐5p	381	878	1141	509				
106	miR‐3559‐3p	153	200	145	156				
107	miR‐375‐3p	328	976	1136	703				
108	miR‐488‐3p	50	232	214	195				
109	miR‐499‐5p	744	1403	1983	1399	219	109	267	162
110	miR‐532‐5p	223	330	463	774	270	218	183	150
111	miR‐674‐3p	466	623	596	388	421	570	445	396
112	miR‐676	217	181	179	178				
113	miR‐7a‐5p	4656	6169	6368	12890	2589	3465	2689	4636
114	miR‐872‐5p	182	319	295	343	238	242	306	408
115	miR‐92a‐3p	140	263	432	444	123	155	246	280
116	miR‐92b‐3p	1594	3159	4246	6237	1291	1603	1919	1733
117	miR‐93‐5p	227	575	307	401	248	335	157	282
118	miR‐98‐5p	1299	2248	2461	2225	1404	1838	1688	2233
119	miR‐99a‐5p	17488	30467	32961	22403	7343	6563	6126	5264
120	miR‐99b‐5p	2020	3320	4289	6080	1305	1212	929	1076
121	miR‐9a‐5p	944	1849	1833	2326	525	507	236	401

**TABLE A2:** Twenty most significantly different miRs carried via EVs.

Number	miR	Log2 fold change	SE (log fold change)	Adjusted *P*‐value
OVX/control vs. Sham/control				
**1**	**miR‐1b**	**−2.958**	**0.371**	**0.000**
**2**	**miR‐378a‐3p**	**−2.259**	**0.402**	**0.000**
**3**	**miR‐133a‐3p**	**−3.333**	**0.595**	**0.000**
**4**	**miR‐100‐5p**	**1.715**	**0.335**	**0.000**
**5**	**miR‐145‐5p**	**−2.574**	**0.579**	**0.000**
**6**	**let‐7g‐5p**	**−0.558**	**0.146**	**0.003**
**7**	**miR‐206‐3p**	**2.237**	**0.668**	**0.014**
**8**	**miR‐30c‐5p**	**−0.538**	**0.178**	**0.037**
**9**	**miR‐146a‐5p**	**−0.900**	**0.302**	**0.039**
10	miR‐221‐3p	1.472	0.538	0.075
11	miR‐155‐5p	1.618	0.686	0.170
12	miR‐182	1.656	0.691	0.170
13	miR‐92b‐3p	0.648	0.274	0.170
14	miR‐183‐5p	1.852	0.810	0.191
15	miR‐31a‐5p	2.079	0.941	0.220
16	miR‐22‐5p	1.789	0.820	0.221
17	miR‐375‐3p	1.282	0.627	0.274
18	miR‐92a‐3p	1.036	0.503	0.274
19	miR‐16‐5p	−0.810	0.415	0.326
20	miR‐30a‐3p	0.810	0.432	0.369
Sham/max vs. Sham/control				
**1**	**miR‐100‐5p**	**2.873**	**0.556**	**0.000**
**2**	**miR‐221‐3p**	**1.782**	**0.526**	**0.035**
**3**	**miR‐127‐3p**	**2.813**	**0.845**	**0.035**
4	miR‐1b	−1.265	0.425	0.086
5	miR‐30c‐5p	−0.544	0.187	0.086
6	miR‐203a‐3p	1.947	0.758	0.162
7	miR‐92b‐3p	0.637	0.250	0.162
8	miR‐31a‐5p	3.005	1.175	0.162
9	miR‐378a‐3p	−1.111	0.450	0.182
10	let‐7g‐5p	−0.388	0.163	0.189
11	miR‐375‐3p	1.482	0.621	0.189
12	miR‐182	1.615	0.787	0.349
13	miR‐206‐3p	1.218	0.594	0.349
14	miR‐7a‐5p	−0.422	0.201	0.349
15	miR‐142‐3p	−0.663	0.349	0.385
16	miR‐143‐3p	−0.418	0.212	0.385
17	miR‐183‐5p	1.662	0.869	0.385
18	miR‐223‐5p	−0.517	0.266	0.385
19	miR‐155‐5p	0.891	0.491	0.415
20	miR‐27a‐3p	0.360	0.196	0.415
OVX/max vs. OVX/control				
**1**	**miR‐122‐5p**	**−1.398**	**0.475**	**0.395**
**2**	**miR‐30e‐3p**	**1.246**	**0.470**	**0.489**
**3**	**miR‐484**	**1.284**	**0.530**	**0.622**
4	miR‐143‐3p	−0.470	0.241	0.782
5	miR‐22‐5p	−1.303	0.670	0.782
6	miR‐141‐3p	1.723	0.892	0.782
7	miR‐133a‐3p	1.448	0.785	0.782
8	miR‐148b‐3p	−0.361	0.197	0.782
9	miR‐145‐5p	−1.221	0.680	0.782
10	miR‐206‐3p	1.575	0.901	0.782
11	miR‐199a‐5p	1.335	0.771	0.782
12	miR‐30c‐5p	0.438	0.257	0.782
13	miR‐451‐5p	−0.638	0.383	0.782
14	miR‐16‐5p	0.649	0.390	0.782
15	miR‐181a‐5p	0.544	0.328	0.782
16	miR‐339‐5p	0.958	0.593	0.802
17	miR‐27b‐3p	0.763	0.515	0.874
18	miR‐218a‐5p	−1.392	0.947	0.874
19	miR‐532‐5p	0.863	0.629	0.874
20	miR‐378a‐3p	0.664	0.486	0.874
OVX/max vs. Sham/max				
**1**	**miR‐145‐5p**	**−2.660**	**0.659**	**0.007**
**2**	**miR‐451‐5p**	**−1.386**	**0.414**	**0.038**
**3**	**miR‐141‐3p**	**2.829**	**0.856**	**0.038**
4	miR‐133a‐3p	−1.663	0.585	0.128
5	miR‐30e‐3p	1.315	0.471	0.128
6	miR‐122‐5p	−1.103	0.416	0.162
7	miR‐1b	−1.085	0.468	0.355
8	miR‐339‐3p	1.129	0.556	0.640
9	miR‐30c‐5p	0.477	0.251	0.772
10	miR‐100‐5p	−0.957	0.552	0.864
11	miR‐1‐3p	1.005	0.656	0.864
12	miR‐151‐3p	−0.240	0.182	0.864
13	miR‐152‐3p	1.142	0.659	0.864
14	miR‐181b‐5p	−0.651	0.453	0.864
15	miR‐200b‐3p	0.690	0.449	0.864
16	miR‐218a‐5p	−1.267	0.924	0.864
17	miR‐23a‐3p	0.746	0.508	0.864
18	miR‐27a‐3p	0.300	0.225	0.864
19	miR‐28‐3p	0.467	0.311	0.864
20	miR‐30e‐5p	0.732	0.457	0.864

*Note*: Statistically significant findings are marked in bold. Abbreviations: miR, microRNA.

**TABLE A3:** Twenty most significantly different miRs carried via HDL particles.

Number	miR	Log2 fold change	SE (log fold change)	Adjusted *P*‐value
OVX/control vs. Sham/control				
**1**	**miR‐182**	**−4.022**	**0.708**	**0.000**
**2**	**miR‐125b‐5p**	**−2.573**	**0.446**	**0.000**
**3**	**miR‐100‐5p**	**−3.468**	**0.634**	**0.000**
**4**	**miR‐221‐3p**	**−1.593**	**0.432**	**0.005**
**5**	**miR‐27b‐3p**	**−1.869**	**0.501**	**0.005**
**6**	**miR‐150‐5p**	**1.336**	**0.381**	**0.008**
**7**	**miR‐21‐5p**	**−0.901**	**0.269**	**0.009**
**8**	**miR‐23a‐3p**	**−1.306**	**0.382**	**0.009**
**9**	**miR‐30a‐5p**	**−1.674**	**0.495**	**0.009**
**10**	**miR‐29a‐3p**	**−1.706**	**0.509**	**0.009**
**11**	**miR‐99b‐5p**	**−1.046**	**0.318**	**0.010**
**12**	**miR‐451‐5p**	**1.439**	**0.470**	**0.019**
**13**	**miR‐103‐3p**	**−1.116**	**0.388**	**0.031**
**14**	**miR‐199a‐3p**	**−2.175**	**0.754**	**0.031**
**15**	**miR‐146b‐5p**	**0.801**	**0.282**	**0.033**
**16**	**miR‐200b‐3p**	**−1.831**	**0.675**	**0.045**
17	miR‐26a‐5p	0.562	0.224	0.076
18	miR‐152‐3p	−1.255	0.514	0.086
19	miR‐122‐5p	1.506	0.622	0.087
20	miR‐22‐3p	0.961	0.423	0.124
Sham/max vs. Sham/control				
**1**	**miR‐30a‐5p**	**−1.912**	**0.524**	**0.028**
**2**	**miR‐100‐5p**	**−2.140**	**0.638**	**0.042**
3	miR‐103‐3p	−0.976	0.348	0.092
4	miR‐27b‐3p	−1.358	0.481	0.092
5	miR‐150‐5p	1.316	0.471	0.092
6	miR‐29a‐3p	−1.484	0.508	0.092
7	miR‐182	−1.418	0.554	0.126
8	miR‐378a‐3p	−1.556	0.606	0.126
9	miR‐99b‐5p	−0.885	0.346	0.126
10	miR‐184	1.688	0.675	0.133
11	miR‐221‐3p	−1.170	0.532	0.268
12	miR‐30a‐3p	−0.806	0.372	0.268
13	miR‐125b‐5p	−1.199	0.574	0.302
14	let‐7b‐5p	0.487	0.248	0.381
15	let‐7c‐5p	0.429	0.249	0.393
16	miR‐125a‐5p	−0.562	0.322	0.393
17	miR‐146a‐5p	0.751	0.397	0.393
18	miR‐152‐3p	−0.978	0.579	0.393
19	miR‐1b	−1.300	0.718	0.393
20	miR‐200b‐3p	−1.111	0.648	0.393
OVX/max vs. OVX/control				
**1**	**miR‐200b‐3p**	**−3.439**	**0.696**	**0.000**
2	miR‐206‐3p	2.394	0.805	0.097
3	miR‐155‐5p	−2.302	0.780	0.097
4	miR‐122‐5p	−1.443	0.496	0.097
5	miR‐143‐3p	−0.943	0.340	0.102
6	miR‐150‐5p	−1.430	0.518	0.102
7	miR‐342‐5p	0.838	0.328	0.148
8	miR‐192‐5p	−1.769	0.715	0.148
9	miR‐223‐5p	0.524	0.213	0.148
10	miR‐27a‐3p	−0.493	0.200	0.148
11	miR‐22‐3p	−0.927	0.395	0.184
12	miR‐7a‐5p	0.591	0.256	0.184
13	let‐7g‐5p	−0.497	0.240	0.313
14	miR‐674‐3p	−0.653	0.330	0.364
15	miR‐184	1.659	0.858	0.370
16	miR‐125a‐5p	0.626	0.328	0.370
17	miR‐99a‐5p	−0.431	0.232	0.370
18	miR‐100‐5p	−0.963	0.522	0.370
19	let‐7d‐3p	0.377	0.207	0.370
20	miR‐26b‐5p	−0.835	0.459	0.370
OVX/max vs. Sham/max				
**1**	**miR‐182**	**−4.022**	**0.708**	**0.000**
**2**	**miR‐125b‐5p**	**−2.573**	**0.446**	**0.000**
**3**	**miR‐100‐5p**	**−3.468**	**0.634**	**0.000**
**4**	**miR‐221‐3p**	**−1.593**	**0.432**	**0.005**
**5**	**miR‐27b‐3p**	**−1.869**	**0.501**	**0.005**
**6**	**miR‐150‐5p**	**1.336**	**0.381**	**0.008**
**7**	**miR‐21‐5p**	**−0.901**	**0.269**	**0.009**
**8**	**miR‐23a‐3p**	**−1.306**	**0.382**	**0.009**
**9**	**miR‐30a‐5p**	**−1.674**	**0.495**	**0.009**
**10**	**miR‐29a‐3p**	**−1.706**	**0.509**	**0.009**
**11**	**miR‐99b‐5p**	**−1.046**	**0.318**	**0.010**
**12**	**miR‐451‐5p**	**1.439**	**0.470**	**0.019**
**13**	**miR‐103‐3p**	**−1.116**	**0.388**	**0.031**
**14**	**miR‐199a‐3p**	**−2.175**	**0.754**	**0.031**
**15**	**miR‐146b‐5p**	**0.801**	**0.282**	**0.033**
**16**	**miR‐200b‐3p**	**−1.831**	**0.675**	**0.045**
17	miR‐26a‐5p	0.562	0.224	0.076
18	miR‐152‐3p	−1.255	0.514	0.086
19	miR‐122‐5p	1.506	0.622	0.087
20	miR‐22‐3p	0.961	0.423	0.124

*Note*: Statistically significant findings are marked in bold. Abbreviation: miR, microRNA.

**TABLE A4:** Twenty most significantly different rDRs carries via HDL particles.

Number	rDR	Annotation	Log2 fold change	SE (log fold change)	Adjusted *P*‐value
OVX/control vs. Sham/control					
1	URS0001B4B318_10116	SSU rRNA	1.929	0.548	0.068
2	URS0001B0949E_10116	SSU rRNA	1.701	0.579	0.255
3	URS000065DC7C_10116	5S rRNA	1.715	0.626	0.319
4	URS00008CB13B_10116	SSU rRNA	1.677	0.645	0.332
5	URS00022B0F36_10116	5S rRNA	1.292	0.506	0.332
6	URS0000623A69_10116	5S rRNA	1.238	0.521	0.453
7	URS00006EFC8C_10116	5S rRNA	1.639	0.707	0.453
8	URS000069C4F1_10116	5S rRNA	0.986	0.459	0.617
9	URS000066C2F6_10116	5S rRNA	0.891	0.438	0.643
10	URS00006CE1FB_10116	5.8S rRNA	0.974	0.487	0.643
11	URS00008C5C0D_10116	SSU rRNA	0.674	0.331	0.643
12	URS0000005270_10116	RNA (157‐MER)(PDB 7QGG. chain D)	0.862	0.492	0.664
13	URS0000070986_10116	Partial rRNA	0.856	0.722	0.664
14	URS00001C1BDA_10116	28S rRNA	0.438	0.300	0.664
15	URS0000315E21_10116	rRNA	0.850	0.499	0.664
16	URS00003E4DE0_10116	Eukaryotic LSU rRNA	0.569	0.444	0.664
17	URS00003ECA53_10116	Eukaryotic LSU rRNA	−0.434	0.379	0.664
18	URS00004213B9_10116	Partial rRNA	0.805	0.679	0.664
19	URS00004B2C76_10116	5.8S rRNA (Rn5‐8s)	0.864	0.499	0.664
20	URS00005296BE_10116	Miscellaneous RNA	0.958	0.677	0.664
Sham/max vs. Sham/control					
1	URS0000005270_10116	RNA (157‐MER)(PDB 7QGG. chain D)	0.211	0.322	0.900
2	URS00000351A9_10116	16S rRNA	0.248	0.419	0.900
3	URS000006A105_10116	16S rRNA	0.430	0.415	0.900
4	URS0000070986_10116	Partial rRNA	−0.242	0.423	0.900
5	URS00000F446C_10116	Partial rRNA	−0.262	0.303	0.900
6	URS00000F8D11_10116	Partial 18S rRNA	−0.236	0.336	0.900
7	URS00000F9D45_10116	RNA (121‐MER)(PDB 7QGG. chain E)	−0.268	0.422	0.900
8	URS0000114737_10116	16S rRNA	−0.403	0.462	0.900
9	URS0000121DBD_10116	Partial rRNA	−0.310	0.292	0.900
10	URS0000129B9F_10116	5S rRNA (variant)	−0.267	0.389	0.900
11	URS00001C1BDA_10116	28S rRNA	0.323	0.263	0.900
12	URS00001CAE00_10116	rRNA	0.347	0.324	0.900
13	URS00001F5E2D_10116	Eukaryotic LSU rRNA	0.269	0.217	0.900
14	URS0000246A92_10116	SSU rRNA	−0.223	0.344	0.900
15	URS0000315E21_10116	rRNA	0.204	0.328	0.900
16	URS000032987A_10116	Partial rRNA	0.225	0.384	0.900
17	URS00003C1820_10116	12S rRNA	0.704	0.512	0.900
18	URS00003E4DE0_10116	Eukaryotic LSU rRNA	−0.154	0.275	0.900
19	URS00003ECA53_10116	Eukaryotic LSU rRNA	−0.289	0.345	0.900
20	URS00003F43F2_10116	5S RNA (Rn5s)	−0.488	0.444	0.900
OVX/max vs. OVX/control					
**1**	**URS00008CB13B_10116**	**SSU rRNA**	**−2.290**	**0.576**	**0.011**
**2**	**URS0001B0949E_10116**	**SSU rRNA**	**−1.863**	**0.526**	**0.031**
**3**	**URS0001B4B318_10116**	**SSU rRNA**	**−1.820**	**0.530**	**0.031**
4	URS00008C5C0D_10116	SSU rRNA	−0.900	0.280	0.051
5	URS00008C5677_10116	SSU rRNA	−0.853	0.303	0.143
6	URS00008C96FC_10116	SSU rRNA	−0.865	0.312	0.143
7	URS00008D1F6F_10116	SSU rRNA	−0.879	0.326	0.157
8	URS00008C644C_10116	SSU rRNA	0.801	0.305	0.165
9	URS00008C6266_10116	SSU rRNA	−0.833	0.324	0.174
10	URS000012BFBF_10116	SSU rRNA	−0.524	0.222	0.279
11	URS00008CAD43_10116	SSU rRNA	−0.829	0.358	0.290
12	URS00005296BE_10116	Miscellaneous RNA	−1.356	0.615	0.328
13	URS00008D160B_10116	SSU rRNA	−0.795	0.358	0.328
14	URS000051C7BA_10116	Eukaryotic LSU rRNA	−0.539	0.254	0.373
15	URS00008C8B6E_10116	SSU rRNA	0.319	0.156	0.419
16	URS00009365D8_10116	Eukaryotic large subunit ribosomal RNA	−1.215	0.610	0.450
17	URS00008C6042_10116	SSU rRNA	0.304	0.163	0.564
18	URS00008C47B3_10116	SSU rRNA	−0.614	0.336	0.585
19	URS00008C6B45_10116	SSU rRNA	−0.221	0.133	0.782
20	URS00000F446C_10116	Partial rRNA	0.376	0.229	0.785
OVX/max vs. Sham/max					
**1**	**URS00000F446C_10116**	**Partial ribosomal RNA**	**0.671**	**0.170**	**0.005**
**2**	**URS00005F96B4_10116**	**SSU rRNA**	**0.559**	**0.143**	**0.005**
**3**	**URS0000121DBD_10116**	**Partial ribosomal RNA**	**0.564**	**0.159**	**0.006**
**4**	**URS000055DB75_10116**	**Partial 18S ribosomal RNA**	**0.661**	**0.184**	**0.006**
**5**	**URS00008C45F1_10116**	**SSU rRNA**	**0.594**	**0.163**	**0.006**
**6**	**URS00008C529B_10116**	**SSU rRNA**	**0.574**	**0.161**	**0.006**
**7**	**URS00008C723E_10116**	**SSU rRNA**	**0.566**	**0.155**	**0.006**
**8**	**URS00001CAE00_10116**	**Ribosomal RNA**	**−0.608**	**0.207**	**0.039**
**9**	**URS00008C5677_10116**	**SSU rRNA**	**−0.868**	**0.297**	**0.039**
**10**	**URS00008C6042_10116**	**SSU rRNA**	**0.408**	**0.140**	**0.039**
**11**	**URS00008C8B6E_10116**	**SSU rRNA**	**0.365**	**0.130**	**0.048**
12	URS00008C59CC_10116	SSU rRNA	0.318	0.129	0.123
13	URS00008C6266_10116	SSU rRNA	−0.663	0.277	0.129
14	URS00008D1F6F_10116	SSU rRNA	−0.804	0.334	0.129
15	URS00008C96FC_10116	SSU rRNA	−0.743	0.329	0.160
16	URS00008CAD43_10116	SSU rRNA	−0.762	0.343	0.160
17	URS00008CE808_10116	SSU rRNA	−0.406	0.182	0.160
18	URS00008D160B_10116	SSU rRNA	−0.762	0.342	0.160
19	URS000012BFBF_10116	SSU rRNA	−0.558	0.260	0.187
20	URS00008C5C0D_10116	SSU rRNA	−0.459	0.230	0.256

*Note*: Statistically significant findings are marked in bold. Abbreviation: rDR, ribosomal RNA (rRNA)‐derived sRNA.

**TABLE A5:** Twenty most significantly different rDRs carries via EVs.

Number	rDR	Annotation	Log2 fold change	SE (log fold change)	Adjusted *P*‐value
OVX/control vs. Sham/control					
1	URS00008CF116_10116	SSU rRNA	−0.788	0.231	0.068
2	URS000069EDAA_10116	5S rRNA	0.755	0.281	0.255
3	URS00008C5677_10116	SSU rRNA	−0.883	0.325	0.319
4	URS00008C59CC_10116	SSU rRNA	−0.419	0.152	0.332
5	URS00008C6042_10116	SSU rRNA	−0.429	0.158	0.332
6	URS00008C644C_10116	rRNA	−0.945	0.318	0.453
7	URS00008C96FC_10116	SSU rRNA	−1.202	0.441	0.453
8	URS00008D1F6F_10116	SSU rRNA	−1.101	0.365	0.617
9	URS000062A825_10116	5S rRNA	0.674	0.315	0.643
10	URS00006600A3_10116	5S rRNA	0.662	0.312	0.643
11	URS00006684C0_10116	5S rRNA	0.692	0.306	0.643
12	URS000066C2F6_10116	5S rRNA	0.647	0.305	0.664
13	URS00006851EC_10116	5S rRNA	0.701	0.278	0.664
14	URS000068E3E4_10116	5.8S rRNA	0.780	0.338	0.664
15	URS000068F5BD_10116	5S rRNA	0.706	0.288	0.664
16	URS000069C4F1_10116	5S rRNA	0.763	0.301	0.664
17	URS00006F3455_10116	5S rRNA	0.678	0.294	0.664
18	URS0000706122_10116	5S rRNA	0.609	0.282	0.664
19	URS0000706CA2_10116	5S rRNA	0.681	0.314	0.664
20	URS00007131E0_10116	5S rRNA	0.676	0.318	0.664
Sham/max vs. Sham/control					
1	URS000065B5CF_10116	5S rRNA (multiple genes)	−0.995	0.308	0.900
2	URS00003F43F2_10116	5S RNA (Rn5s)	−0.934	0.316	0.900
3	URS0000CD0331_10116	5S RNA (Rn5s)	−0.957	0.333	0.900
4	URS00006E79BE_10116	5.8S rRNA	−0.943	0.383	0.900
5	URS00006EBFDA_10116	5.8S rRNA	−0.915	0.352	0.900
6	URS00008C6266_10116	SSU rRNA	0.924	0.348	0.900
7	URS00008D160B_10116	SSU rRNA	0.816	0.315	0.900
8	URS00022B3C24_10116	5S rRNA	−0.893	0.357	0.900
9	URS00008D1F6F_10116	SSU rRNA	0.935	0.389	0.900
10	URS00022B91B4_10116	5S rRNA	−0.907	0.381	0.900
11	URS00000F9D45_10116	RNA (121‐MER) (PDB 7QGG. chain E)	−0.915	0.484	0.900
12	URS0000184DE3_10116	5S rRNA	−0.979	0.453	0.900
13	URS00001F5E2D_10116	Eukaryotic LSU rRNA	0.393	0.214	0.900
14	URS0000246A92_10116	SSU rRNA	0.387	0.214	0.900
15	URS000032987A_10116	Partial rRNA	−0.508	0.298	0.900
16	URS000051C7BA_10116	Eukaryotic LSU rRNA	0.276	0.162	0.900
17	URS00005296BE_10116	Miscellaneous RNA	−0.927	0.445	0.900
18	URS0000629A3C_10116	5.8S rRNA	−0.722	0.434	0.900
19	URS000062C46B_10116	5.8S rRNA	−0.539	0.283	0.900
20	URS0000637711_10116	5S rRNA	−0.719	0.414	0.900
OVX/max vs. OVX/control					
**1**	**URS00008C96FC_10116**	**SSU rRNA**	**0.930**	**0.276**	**0.011**
**2**	**URS000068F5BD_10116**	**5S rRNA**	**−0.758**	**0.274**	**0.031**
**3**	**URS00006F129F_10116**	**5S rRNA**	**−0.677**	**0.267**	**0.031**
4	URS0000706122_10116	5S rRNA	−0.798	0.300	0.051
5	URS00008C5677_10116	SSU rRNA	0.701	0.271	0.143
6	URS00008D1F6F_10116	SSU rRNA	0.771	0.303	0.143
7	URS00022B53F0_10116	5S rRNA	−0.759	0.288	0.157
8	URS00022B8935_10116	5S rRNA	−0.768	0.289	0.165
9	URS000012BFBF_10116	SSU rRNA	0.452	0.186	0.174
10	URS0000548AD6_10116	12S rRNA	−0.964	0.392	0.279
11	URS00006E79BE_10116	5.8S rRNA	−0.795	0.333	0.290
12	URS0000647009_10116	5S rRNA	−0.684	0.290	0.328
13	URS00005296BE_10116	Miscellaneous RNA	1.597	0.689	0.328
14	URS00006E6F77_10116	5S rRNA (multiple genes)	−0.667	0.299	0.373
15	URS00008CB5BD_10116	SSU rRNA	0.428	0.196	0.419
16	URS00022B6B03_10116	5S rRNA	−0.590	0.272	0.450
17	URS0000693ECA_10116	5S rRNA	−0.573	0.273	0.564
18	URS00001F5E2D_10116	Eukaryotic LSU rRNA	0.439	0.229	0.585
19	URS000064596E_10116	5S rRNA	−0.572	0.296	0.782
20	URS000065DC7C_10116	5S rRNA	−0.626	0.309	0.785
OVX/max vs. Sham/max					
1	URS00001D9CDD_10116	16S ribosomal RNA	−1.322	0.387	0.129
2	URS0000114737_10116	16S ribosomal RNA	−1.274	0.400	0.148
3	URS00001AFCBA_10116	16S ribosomal RNA	−1.020	0.399	0.290
4	URS00001E528E_10116	Large subunit ribosomal RNA	−1.015	0.414	0.290
5	URS00001F5E2D_10116	Eukaryotic LSU rRNA	0.469	0.192	0.290
6	URS000041298D_10116	16S ribosomal RNA	−0.927	0.393	0.290
7	URS000045493D_10116	Large subunit ribosomal RNA	−0.899	0.357	0.290
8	URS0000548AD6_10116	12S ribosomal RNA	−1.089	0.448	0.290
9	URS000055DB75_10116	Partial 18S ribosomal RNA	−0.431	0.185	0.290
10	URS00005F96B4_10116	SSU rRNA	−0.381	0.163	0.290
11	URS00007D4C97_10116	16S ribosomal RNA	−0.945	0.403	0.290
12	URS00007D6E2A_10116	12S ribosomal RNA	−1.175	0.439	0.290
13	URS000091179F_10116	Bacterial large subunit ribosomal RNA	−1.111	0.423	0.290
14	URS0002643043_10116	16S ribosomal RNA	−1.070	0.397	0.290
15	URS00000382E0_10116	12S rRNA	−0.865	0.413	0.291
16	URS00003ECA53_10116	Eukaryotic LSU rRNA	0.559	0.264	0.291
17	URS00008C5677_10116	SSU rRNA	0.635	0.287	0.291
18	URS00008C96FC_10116	SSU rRNA	0.679	0.301	0.291
19	URS00008CB01C_10116	Eukaryotic LSU rRNA	0.453	0.218	0.291
20	URS00008CBDB7_10116	Eukaryotic LSU rRNA	0.443	0.212	0.291

*Note*: Statistically significant findings are marked in bold. Abbreviation: rDR, ribosomal RNA (rRNA)‐derived sRNA.

**TABLE A6:** Twenty most significantly different tDRs carried via EVs.

Number	tDR	Log2 fold change	SE (log fold change)	Adjusted *P*‐value
OVX/control vs. Sham/control				
**1**	**tRNA‐Met‐CAT‐3‐1**	**4.196**	**0.699**	**0.000**
**2**	**tRNA‐Met‐CAT‐1‐1**	**3.361**	**0.652**	**0.000**
3	tRNA‐Ala‐TGC‐1‐1	0.799	0.412	0.654
4	tRNA‐Ala‐TGC‐1‐2	0.733	0.455	0.654
5	tRNA‐Arg‐CCT‐3‐1	1.101	0.565	0.654
6	tRNA‐Arg‐CCT‐4‐1	1.073	0.565	0.654
7	tRNA‐Asp‐GTC‐1‐1	−0.997	0.430	0.654
8	tRNA‐Glu‐CTC‐1‐10	−0.712	0.407	0.654
9	tRNA‐Glu‐CTC‐1‐11	−0.711	0.412	0.654
10	tRNA‐Glu‐CTC‐1‐12	−0.674	0.414	0.654
11	tRNA‐Glu‐CTC‐1‐2	−0.712	0.410	0.654
12	tRNA‐Glu‐CTC‐1‐3	−0.684	0.406	0.654
13	tRNA‐Glu‐CTC‐1‐4	−0.689	0.411	0.654
14	tRNA‐Glu‐CTC‐1‐7	−0.690	0.413	0.654
15	tRNA‐Glu‐CTC‐1‐9	−0.729	0.415	0.654
16	tRNA‐Glu‐CTC‐2‐1	0.525	0.296	0.654
17	tRNA‐Glu‐CTC‐4‐1	−0.678	0.409	0.654
18	tRNA‐Glu‐TTC‐2‐1	−0.588	0.324	0.654
19	tRNA‐Glu‐TTC‐3‐1	−0.484	0.303	0.654
20	tRNA‐Lys‐CTT‐1‐4	−0.630	0.313	0.654
Sham/max vs. Sham/control				
**1**	**tRNA‐Met‐CAT‐3‐1**	**3.802**	**0.706**	**0.000**
**2**	tRNA‐Leu‐AAG‐2‐1	2.458	0.739	0.058
3	tRNA‐Met‐CAT‐1‐1	2.418	0.734	0.058
4	tRNA‐Leu‐AAG‐2‐2	2.231	0.734	0.084
5	tRNA‐Leu‐AAG‐2‐3	2.275	0.742	0.084
6	tRNA‐Glu‐CTC‐1‐1	−0.887	0.419	0.332
7	tRNA‐Glu‐CTC‐1‐10	−0.924	0.418	0.332
8	tRNA‐Glu‐CTC‐1‐11	−0.984	0.418	0.332
9	tRNA‐Glu‐CTC‐1‐2	−0.875	0.416	0.332
10	tRNA‐Glu‐CTC‐1‐3	−0.914	0.418	0.332
11	tRNA‐Glu‐CTC‐1‐6	−0.886	0.419	0.332
12	tRNA‐Glu‐CTC‐1‐7	−0.905	0.422	0.332
13	tRNA‐Glu‐CTC‐1‐8	−0.923	0.426	0.332
14	tRNA‐Glu‐CTC‐1‐9	−0.903	0.420	0.332
15	tRNA‐Glu‐CTC‐4‐1	−0.901	0.417	0.332
16	tRNA‐Leu‐CAA‐1‐1	1.311	0.571	0.332
17	tRNA‐Leu‐CAA‐2‐1	1.355	0.566	0.332
18	tRNA‐Ser‐AGA‐3‐1	1.323	0.531	0.332
19	tRNA‐Ser‐GCT‐3‐4	1.189	0.534	0.332
20	tRNA‐Glu‐CTC‐1‐12	−0.874	0.420	0.332
OVX/max vs. OVX/control				
1	tRNA‐Ala‐CGC‐3‐1	0.953	0.414	0.632
2	tRNA‐Glu‐TTC‐2‐2	−0.575	0.244	0.632
3	tRNA‐Glu‐TTC‐3‐1	−0.479	0.201	0.632
4	tRNA‐Gly‐CCC‐3‐1	−1.082	0.459	0.632
5	tRNA‐Lys‐TTT‐4‐1	1.633	0.636	0.632
6	tRNA‐Pro‐CGG‐1‐1	0.526	0.238	0.632
7	tRNA‐Pro‐TGG‐2‐1	1.161	0.530	0.632
8	tRNA‐SeC‐TCA‐1‐1	−1.431	0.526	0.632
9	tRNA‐Ala‐AGC‐1‐1	0.800	0.433	0.669
10	tRNA‐Ala‐AGC‐2‐1	0.783	0.553	0.669
11	tRNA‐Ala‐AGC‐2‐2	0.832	0.555	0.669
12	tRNA‐Ala‐AGC‐3‐1	0.676	0.546	0.669
13	tRNA‐Ala‐AGC‐3‐3	0.879	0.550	0.669
14	tRNA‐Ala‐CGC‐1‐1	0.857	0.469	0.669
15	tRNA‐Ala‐CGC‐2‐1	0.670	0.403	0.669
16	tRNA‐Ala‐TGC‐1‐1	0.464	0.426	0.669
17	tRNA‐Ala‐TGC‐2‐1	0.484	0.402	0.669
18	tRNA‐Ala‐TGC‐3‐1	0.811	0.556	0.669
19	tRNA‐Ala‐TGC‐4‐1	0.727	0.588	0.669
20	tRNA‐Arg‐CCT‐3‐1	−0.433	0.414	0.669
OVX/max vs. Sham/max				
**1**	**tRNA‐Met‐CAT‐3‐1**	**4.196**	**0.699**	**0.000**
**2**	**tRNA‐Met‐CAT‐1‐1**	**3.361**	**0.652**	**0.000**
3	tRNA‐Ala‐TGC‐1‐1	0.799	0.412	0.654
4	tRNA‐Ala‐TGC‐1‐2	0.733	0.455	0.654
5	tRNA‐Arg‐CCT‐3‐1	1.101	0.565	0.654
6	tRNA‐Arg‐CCT‐4‐1	1.073	0.565	0.654
7	tRNA‐Asp‐GTC‐1‐1	−0.997	0.430	0.654
8	tRNA‐Glu‐CTC‐1‐10	−0.712	0.407	0.654
9	tRNA‐Glu‐CTC‐1‐11	−0.711	0.412	0.654
10	tRNA‐Glu‐CTC‐1‐12	−0.674	0.414	0.654
11	tRNA‐Glu‐CTC‐1‐2	−0.712	0.410	0.654
12	tRNA‐Glu‐CTC‐1‐3	−0.684	0.406	0.654
13	tRNA‐Glu‐CTC‐1‐4	−0.689	0.411	0.654
14	tRNA‐Glu‐CTC‐1‐7	−0.690	0.413	0.654
15	tRNA‐Glu‐CTC‐1‐9	−0.729	0.415	0.654
16	tRNA‐Glu‐CTC‐2‐1	0.525	0.296	0.654
17	tRNA‐Glu‐CTC‐4‐1	−0.678	0.409	0.654
18	tRNA‐Glu‐TTC‐2‐1	−0.588	0.324	0.654
19	tRNA‐Glu‐TTC‐3‐1	−0.484	0.303	0.654
20	tRNA‐Lys‐CTT‐1‐4	−0.630	0.313	0.654

*Note*: Statistically significant findings are marked in bold. Abbreviation: tDR, transfer RNA (tRNA)‐derived sRNA.

**TABLE A7:** Twenty most significantly different tDRs carried via HDL particles.

Number	tDR	Log2 fold change	SE (log fold change)	Adjusted *P*‐value
OVX/control vs. Sham/control				
1	tRNA‐Glu‐TTC‐2‐2	1.012	0.411	0.306
2	tRNA‐Gly‐GCC‐2‐1	−0.426	0.167	0.306
3	tRNA‐Gly‐GCC‐2‐6	−0.398	0.153	0.306
4	tRNA‐Gly‐GCC‐4‐1	−0.383	0.131	0.306
5	tRNA‐His‐GTG‐1‐2	−1.158	0.478	0.306
6	tRNA‐His‐GTG‐1‐3	−1.195	0.466	0.306
7	tRNA‐His‐GTG‐1‐5	−1.211	0.484	0.306
8	tRNA‐His‐GTG‐1‐8	−1.211	0.472	0.306
9	tRNA‐Ala‐AGC‐1‐1	1.518	0.706	0.425
10	tRNA‐Glu‐TTC‐2‐1	0.868	0.403	0.425
11	tRNA‐Glu‐TTC‐3‐1	0.887	0.414	0.425
12	tRNA‐Gly‐GCC‐2‐2	−0.367	0.165	0.425
13	tRNA‐Gly‐GCC‐2‐3	−0.354	0.168	0.425
14	tRNA‐Gly‐GCC‐2‐4	−0.383	0.190	0.440
15	tRNA‐Gly‐GCC‐2‐5	−0.331	0.165	0.440
16	tRNA‐His‐GTG‐1‐10	−1.049	0.519	0.440
17	tRNA‐His‐GTG‐1‐7	−0.964	0.485	0.440
18	tRNA‐His‐GTG‐1‐11	−0.934	0.481	0.443
19	tRNA‐Pro‐CGG‐1‐1	−0.786	0.406	0.443
20	tRNA‐Gln‐CTG‐1‐1	0.997	0.538	0.509
Sham/max vs. Sham/control				
1	tRNA‐Arg‐CCG‐3‐1	−3.095	0.991	0.265
2	tRNA‐Pro‐CGG‐1‐1	−1.281	0.437	0.265
3	tRNA‐Ala‐AGC‐1‐1	1.261	0.701	0.968
4	tRNA‐Ala‐AGC‐2‐2	0.465	0.576	0.968
5	tRNA‐Ala‐AGC‐3‐1	0.555	0.605	0.968
6	tRNA‐Ala‐AGC‐3‐3	0.658	0.640	0.968
7	tRNA‐Ala‐CGC‐1‐1	0.971	0.743	0.968
8	tRNA‐Ala‐CGC‐2‐1	0.599	0.678	0.968
9	tRNA‐Ala‐CGC‐3‐1	0.621	0.713	0.968
10	tRNA‐Ala‐TGC‐1‐1	0.614	0.690	0.968
11	tRNA‐Ala‐TGC‐1‐2	0.696	0.671	0.968
12	tRNA‐Ala‐TGC‐2‐1	0.882	0.798	0.968
13	tRNA‐Ala‐TGC‐3‐1	0.482	0.597	0.968
14	tRNA‐Ala‐TGC‐4‐1	0.726	0.751	0.968
15	tRNA‐Asp‐GTC‐2‐11	0.437	0.534	0.968
16	tRNA‐Asp‐GTC‐2‐14	0.586	0.650	0.968
17	tRNA‐Asp‐GTC‐2‐5	0.552	0.595	0.968
18	tRNA‐Cys‐GCA‐1‐1	0.414	0.488	0.968
19	tRNA‐Gln‐CTG‐1‐1	0.481	0.448	0.968
20	tRNA‐Gln‐CTG‐1‐2	0.437	0.462	0.968
OVX/max vs. OVX/control				
1	tRNA‐Leu‐AAG‐2‐1	−1.988	0.639	0.297
2	tRNA‐Leu‐AAG‐2‐3	−1.714	0.594	0.309
3	tRNA‐Gln‐CTG‐7‐1	−1.511	0.598	0.608
4	tRNA‐Ala‐AGC‐1‐1	−0.524	0.571	0.987
5	tRNA‐Ala‐CGC‐1‐1	−0.492	0.565	0.987
6	tRNA‐Ala‐CGC‐2‐1	−0.957	0.560	0.987
7	tRNA‐Ala‐CGC‐3‐1	−0.298	0.522	0.987
8	tRNA‐Ala‐TGC‐1‐1	−0.891	0.610	0.987
9	tRNA‐Ala‐TGC‐1‐2	−0.942	0.608	0.987
10	tRNA‐Ala‐TGC‐2‐1	−0.612	0.583	0.987
11	tRNA‐Ala‐TGC‐3‐1	0.292	0.501	0.987
12	tRNA‐Ala‐TGC‐4‐1	−0.711	0.619	0.987
13	tRNA‐Arg‐CCG‐3‐1	−0.875	0.720	0.987
14	tRNA‐Asp‐GTC‐2‐2	0.417	0.439	0.987
15	tRNA‐Cys‐GCA‐11‐1	−0.924	0.567	0.987
16	tRNA‐Cys‐GCA‐1‐3	0.309	0.420	0.987
17	tRNA‐Cys‐GCA‐1‐4	0.459	0.438	0.987
18	tRNA‐Cys‐GCA‐2‐1	0.406	0.454	0.987
19	tRNA‐Cys‐GCA‐2‐2	0.554	0.472	0.987
20	tRNA‐Gln‐CTG‐1‐1	−0.426	0.423	0.987
OVX/max vs. Sham/max				
1	tRNA‐Glu‐TTC‐2‐2	1.012	0.411	0.306
2	tRNA‐Gly‐GCC‐2‐1	−0.426	0.167	0.306
3	tRNA‐Gly‐GCC‐2‐6	−0.398	0.153	0.306
4	tRNA‐Gly‐GCC‐4‐1	−0.383	0.131	0.306
5	tRNA‐His‐GTG‐1‐2	−1.158	0.478	0.306
6	tRNA‐His‐GTG‐1‐3	−1.195	0.466	0.306
7	tRNA‐His‐GTG‐1‐5	−1.211	0.484	0.306
8	tRNA‐His‐GTG‐1‐8	−1.211	0.472	0.306
9	tRNA‐Ala‐AGC‐1‐1	1.518	0.706	0.425
10	tRNA‐Glu‐TTC‐2‐1	0.868	0.403	0.425
11	tRNA‐Glu‐TTC‐3‐1	0.887	0.414	0.425
12	tRNA‐Gly‐GCC‐2‐2	−0.367	0.165	0.425
13	tRNA‐Gly‐GCC‐2‐3	−0.354	0.168	0.425
14	tRNA‐Gly‐GCC‐2‐4	−0.383	0.190	0.440
15	tRNA‐Gly‐GCC‐2‐5	−0.331	0.165	0.440
16	tRNA‐His‐GTG‐1‐10	−1.049	0.519	0.440
17	tRNA‐His‐GTG‐1‐7	−0.964	0.485	0.440
18	tRNA‐His‐GTG‐1‐11	−0.934	0.481	0.443
19	tRNA‐Pro‐CGG‐1‐1	−0.786	0.406	0.443
20	tRNA‐Gln‐CTG‐1‐1	0.997	0.538	0.509

*Note*: Statistically significant findings are marked in bold. Abbreviations: tDR, transfer RNA (tRNA)‐derived sRNA.

**TABLE A8:** Significant Kyoto Encyclopedia of Genes and Genomes (KEGG) pathways of significantly different miRs carried via EVs.

Number	KEGG pathway	*P*	Number of regulated genes	Number of regulating miRs
OVX/control vs. Sham/control				
1	Proteoglycans in cancer	0.038	2	1
2	Type II diabetes mellitus	0.057	4	3
Sham/max vs. Sham/control				
1	Adipocytokine signalling pathway	0.025	1	1
2	ErbB signalling pathway	0.030	1	1
3	Wnt signalling pathway	0.032	1	1
4	MicroRNAs in cancer	0.035	2	1
5	HTLV‐I infection	0.039	1	1
6	Caffeine metabolism	0.039	1	1
7	Pathways in cancer	0.040	3	1
8	Proteoglycans in cancer	0.046	2	1
9	MAPK signalling pathway	0.051	1	1
10	HIF‐1 signalling pathway	0.052	1	1

**TABLE A9:** Significant Kyoto Encyclopedia of Genes and Genomes (KEGG) pathways of significantly different miRs carried via HDL particles.

Number	KEGG pathway	*P*	Number of regulated genes	Number of regulating miRs
OVX/control vs. Sham/control				
1	MicroRNAs in cancer	0.003	2	2
2	Proteoglycans in cancer	0.014	2	2
3	PI3K‐Akt signalling pathway	0.016	2	2
4	Pathways in cancer	0.019	3	2
5	Endocytosis	0.030	5	3
6	MAPK signalling pathway	0.031	5	3
7	HIF‐1 signalling pathway	0.054	2	2
Sham/max vs. Sham/control				
1	Type II diabetes mellitus	0.002	1	1
2	Central carbon metabolism in cancer	0.003	2	1
3	Glycosaminoglycan biosynthesis—heparan sulfate / heparin	0.003	1	1
4	Proteoglycans in cancer	0.006	2	1
5	ErbB signalling pathway	0.008	1	1
6	mTOR signalling pathway	0.009	1	1
7	Adipocytokine signalling pathway	0.010	1	1
8	HIF‐1 signalling pathway	0.011	1	1
9	Signaling pathways regulating pluripotency of stem cells	0.013	2	1
10	MicroRNAs in cancer	0.013	2	1
11	Glioma	0.014	1	1
12	Choline metabolism in cancer	0.016	1	1
13	Basal cell carcinoma	0.017	1	1
14	Pathways in cancer	0.017	3	1
15	Acute myeloid leukemia	0.017	1	1
16	Insulin signalling pathway	0.019	1	1
17	Rap1 signalling pathway	0.020	1	1
18	Endocytosis	0.022	1	1
19	Regulation of actin cytoskeleton	0.023	1	1
20	Thyroid hormone signalling pathway	0.024	1	1
21	Melanogenesis	0.026	1	1
22	AMPK signalling pathway	0.026	1	1
23	Bladder cancer	0.026	1	1
24	HTLV‐I infection	0.030	1	1
25	PI3K‐Akt signalling pathway	0.030	2	1
26	Wnt signalling pathway	0.031	1	1
27	Prostate cancer	0.034	1	1
28	Ras signalling pathway	0.042	1	1
29	Hippo signalling pathway	0.044	1	1
30	MAPK signalling pathway	0.047	1	1
OVX/max vs. Sham/max				
1	Mucin type O‐Glycan biosynthesis	0.000	5	3
2	Proteoglycans in cancer	0.000	18	3
3	Fatty acid degradation	0.000	1	2
4	MicroRNAs in cancer	0.001	14	3
5	N‐Glycan biosynthesis	0.001	6	2
6	Glycosaminoglycan biosynthesis—keratan sulfate	0.001	2	4
7	ECM‐receptor interaction	0.007	4	2
8	Transcriptional misregulation in cancer	0.021	18	2
9	Glycosaminoglycan biosynthesis—chondroitin sulfate / dermatan sulfate	0.038	2	2
10	MAPK signalling pathway	0.056	17	1
